# Structural and functional insights into TBC1D17 highlight the importance of the previously uncharacterized Rab‐binding domain

**DOI:** 10.1002/pro.70581

**Published:** 2026-04-17

**Authors:** Dominika Nielipińska, Marta Orlikowska, Maciej Nielipiński, Bartosz Sekuła, Katarzyna M. Błażewska, Edyta Gendaszewska‐Darmach, Agnieszka J. Pietrzyk‐Brzezińska

**Affiliations:** ^1^ Institute of Molecular and Industrial Biotechnology, Faculty of Biotechnology and Food Sciences Lodz University of Technology Lodz Poland; ^2^ Department of Biomedical Chemistry, Faculty of Chemistry University of Gdansk Gdansk Poland; ^3^ Institute of Organic Chemistry, Faculty of Chemistry Lodz University of Technology Lodz Poland

**Keywords:** GTPase activating protein, Rab‐GAP interactions, TBC1D17 protein, type 2 diabetes

## Abstract

TBC (Tre2/Bub2/Cdc16) domain‐containing proteins constitute the widespread family of GTPase‐activating proteins (GAPs). They interact with the Rab superfamily of small GTPases, stimulate GTP hydrolysis, and regulate vesicle trafficking. TBC1D17, involved in Shiga toxin trafficking, autophagy and glucose metabolism regulation, constitutes an example of GAP interacting with Rabs. Here we present the first crystal structures of the murine and human TBC domains of TBC1D17 proteins determined at 2.20 and 3.34 Å resolution, respectively. The TBC domain in both structures represents a heart‐like shape. Our analyses revealed dimerization of the TBC domain through a fragment located near residues participating in GTP hydrolysis, a result we observed also in structures of closely related homologs. Furthermore, we tested Rab5a interactions with various fragments of TBC1D17. Interestingly, this protein contains an annotated, yet uncharacterized, Rab‐binding domain (RBD) and our studies revealed strong interactions of Rab5a with TBC1D17 fragments containing RBD, while interactions with the TBC domain alone are much weaker. These results provide the first direct evidence for the critical role of the TBC1D17 RBD in interactions with Rab5a.

## 1 INTRODUCTION

Vesicle trafficking is a fundamental process, enabling transport of cargo from the cell surface, inside or outside of the cell, and between organelles. In the endocytic pathway, the cargo constitutes nutrients, metabolites, proteins, signaling molecules, fragments of the cellular membrane, or other components. These compounds are enclosed in double‐membrane structures called primary endocytic vesicles, which undergo fusion with early endosomes (EE) (Zhang et al., 2022). In EEs, a preliminary sorting of the cargo takes place, and the cargo is directed back to the cell membrane for reuse or to further transport into the cell interior. Along with transport near the nucleus, EEs are getting bigger and their composition changes, leading to formation of late endosomes (LE), where a second cycle of recycling is conducted. The last stage of maturation includes fusion of LE with lysosomes and degradation of cargo (Jeger, 2020).

Every step of maturation, as well as transport and fusion of vesicles with the cell membrane, is controlled by Rab GTPases (Wandinger‐Ness & Zerial, 2014). These proteins constitute the largest subfamily of small G proteins within the Ras superfamily with over 60 representatives identified in the human genome (Gendaszewska‐Darmach et al., 2021). Their GTP‐dependent activity and the nucleotide exchange are tightly controlled by regulatory proteins. Newly synthesized Rabs circulating in the cytoplasm in their GDP‐bound forms are recruited by Rab escort protein (REP) and subsequently undergo posttranslational modification catalyzed by Rab prenyltransferases (Pylypenko et al., 2018). They attach one or two geranylgeranyl or farnesyl groups to cysteine residues located at the Rab C‐terminus, enabling the protein anchoring to organelle membranes. Once incorporated, Rab interacts with guanine nucleotide exchange factor (GEF) which promotes GDP release from the active site and creation of Rab‐GTP complex. Once activated, the protein recruits effectors (e.g., AKAP10, Rabaptin5, RILP interacting with Rab4, Rab5 and Rab7, respectively) and fulfills specific cellular functions such as transferrin receptor recycling, endosome maturation, and LE‐to‐lysosome trafficking (Zhang et al., 2022). Then Rab is inactivated through GTP hydrolysis, stimulated by interaction with GTPase activating protein (GAP). The cycle is completed by guanine GDP dissociation inhibitor (GDI), which extracts the Rab‐GDP complex from the membrane and directs it for the subsequent round of activity (Pylypenko et al., 2018).

Up to now, over 40 different GAPs containing a Tre‐2/Bub2/Cdc16 (TBC) domain have been identified, with the TBC1D17 protein serving as a representative member of this group. According to Frasa et al., TBC1D17 belongs to conventional GAPs, as its mechanism of action involves arginine and glutamine residues located in highly conserved IxxDxxR and YxQ motifs (Frasa et al., 2012; Pan et al., 2006). The first interaction partners of TBC1D17, Rab5a/b/c, have been identified in yeast two‐hybrid screening (Itoh et al., 2006). In vitro, TBC1D17 exhibited GAP activity towards several Rab GTPases other than Rab5a‐c (including Rab1, Rab8a, Rab13, Rab21, and Rab35), suggesting a broad substrate spectrum (Fuchs et al., 2007). In HeLa cells, it was specifically implicated in restricting Shiga toxin trafficking, although the precise Rab substrate mediating this effect remained unresolved in the study (Fuchs et al., 2007). It is of note that, in addition to the stimulation of GTP hydrolysis in small GTPases, TBC1D17, similarly to other GAPs containing the TBC domain, participates in the regulation of some processes, like autophagy, via interactions with other proteins (Yamano et al., 2014).

It was later observed that the interaction between TBC1D17 and Rab8 requires the adaptor protein optineurin, a receptor of autophagy involved in the proper functioning of the Golgi apparatus and vesicle trafficking (Ryan & Tumbarello, 2018). Together, they form a ternary complex that inhibits endocytosis and recycling of the transferrin receptor (Vaibhava et al., 2012; Zhang et al., 2024). Moreover, TBC1D17 can form homodimers or heterodimers (with TBC1D15), which in turn bind dimeric mitochondrial fission protein 1 (Fis1). Through negative regulation of Rab7, together with Fis1, TBC1D17 regulates autophagosome formation and prevents their excessive formation (Yamano et al., 2014). Interestingly, through the LC3‐interacting region (LIR) motif, TBC1D17 also interacts with representatives of autophagy‐related proteins 8 (Atg8)—members of GABARAP and LC3 subfamilies, which are recognized by adaptor proteins such as optineurin and engulf mitochondria in the phagophore during Parkin‐dependent mitophagy (Eshraghi et al., 2021; Yamano et al., 2014). In addition, under hypoxic stress in the Parkin‐deprived HeLa cell line, the formation of the Fis1‐TBC1D17 dimer depends on SUMOylation of Fis1 and such interaction inhibits mitophagy (Zhao et al., 2024). Finally, Rao et al. (2021) confirmed the GAP activity of TBC1D17 towards Rab5a mediated by its TBC domain (307–542). GAP activity was assessed in vivo in C2C12 myoblasts and in vitro in the GAP assay, while direct interaction between Rab5a and the TBC domain (307–542) was detected using 293T cells (Rao et al., 2021). Interestingly, they observed more significant inactivation of Rab5a by the TBC domain than by full‐length TBC1D17 protein. This effect is explained by the direct interaction of AMP‐activated kinase (AMPK) α1‐ and α2‐subunits, sensors of ATP/ADP/AMP in skeletal muscles and other tissues (Hardie et al., 2012), with the N‐terminal domain (1–306) of TBC1D17. The authors of the study suggested that phosphorylation on this position induces conformational changes promoting stronger interaction between the TBC1D17 N‐terminal part and the TBC domain, which might cause the covering of residues responsible for the acceleration of GTP hydrolysis by the Rab5a protein (Rao et al., 2021). AMPK phosphorylates Ser_168_ of TBC1D17 and leads to protein autoinhibition. This mechanism is particularly important because active TBC1D17 inhibits the transport of glucose transporters type 4 (GLUT4) and type 1 (GLUT1) to the cell membrane and reduces glucose uptake, as observed in the C2C12 cell line (Rao et al., 2021).

Knowledge of the three‐dimensional structure of proteins and their complexes enables rational design of compounds with high potential for specific interaction (Ouma et al., 2024). It is especially useful for synthesis of protein–protein interaction (PPI) inhibitors. Up to now, many small molecules, peptides, and peptidomimetics specific towards disease‐related molecular targets have been synthesized (Nada et al., 2024). The most common targets constitute proteins involved in the progression of cancer, immunological, and viral diseases (Lu et al., 2020). Many studies are also devoted to the development of antidiabetic compounds. Given the widespread global incidence of this metabolic disease (590 million adults worldwide aged 20–79 in 2025, with 90% cases being type 2 diabetes (T2D) according to the International Diabetes Federation (2025)), many medications have been developed and approved. These include synthetic small molecules as well as drugs designed based on natural ligands or agonists of proteins engaged in glucose metabolism (Bailey et al., 2025; Guo et al., 2024). Moreover, development of novel therapeutic strategies such as stapled peptides and PROTACs, targeting PPIs or directing proteins to degradation, respectively, represent promising avenues for T2D treatment (Nielipińska et al., 2024; Sobierajski et al., 2024). Genetic and physiological studies demonstrate that TBC1D1 and TBC1D4 regulate GLUT4 translocation and glucose uptake in muscle, reinforcing the relevance of RabGAP‐controlled trafficking to metabolic control (Cartee, 2015). In the light of reports on down‐regulation of Rabs in diabetes, reviewed by Gendaszewska‐Darmach et al. (2021), and multiple examples identifying pairs of Rab proteins and GAPs, as involved in insulin‐induced GLUT4 translocation, we focused here on the recently characterized Rab5‐TBC1D17 axis, which modulates GLUT trafficking in myoblasts and skeletal muscle (Rao et al., 2021). Considering TBC1D17 involvement in glucose metabolism, we decided to crystallize and solve the structure of its TBC domain as a potential target for drug development. It is of note that TBC1D17 has not previously been structurally characterized, and the structures reported here represent the first experimental structural data for this protein. We determined the structures of murine and human TBC1D17 which are biologically relevant for utilization in drug development process. We analyzed the structural features and sequence conservation. We also focused on the N‐terminal fragment of TBC1D17. To date, this part of the protein has not been characterized. The only report concerning this part of the protein described, as mentioned earlier, that AMPK phosphorylates Ser_168_ of TBC1D17 and leads to protein autoinhibition which indicates that the N‐terminal part of TBC1D17 might also play an important role. Interestingly, a large part of it is annotated in the InterPro database as a Rab‐binding domain (RBD). Our biochemical characterization of this domain confirmed its engagement in interaction with Rab5a.

## 2 RESULTS

### 2.1 Structures of the TBC domain of mammalian TBC1D17 proteins

**FIGURE 1:**
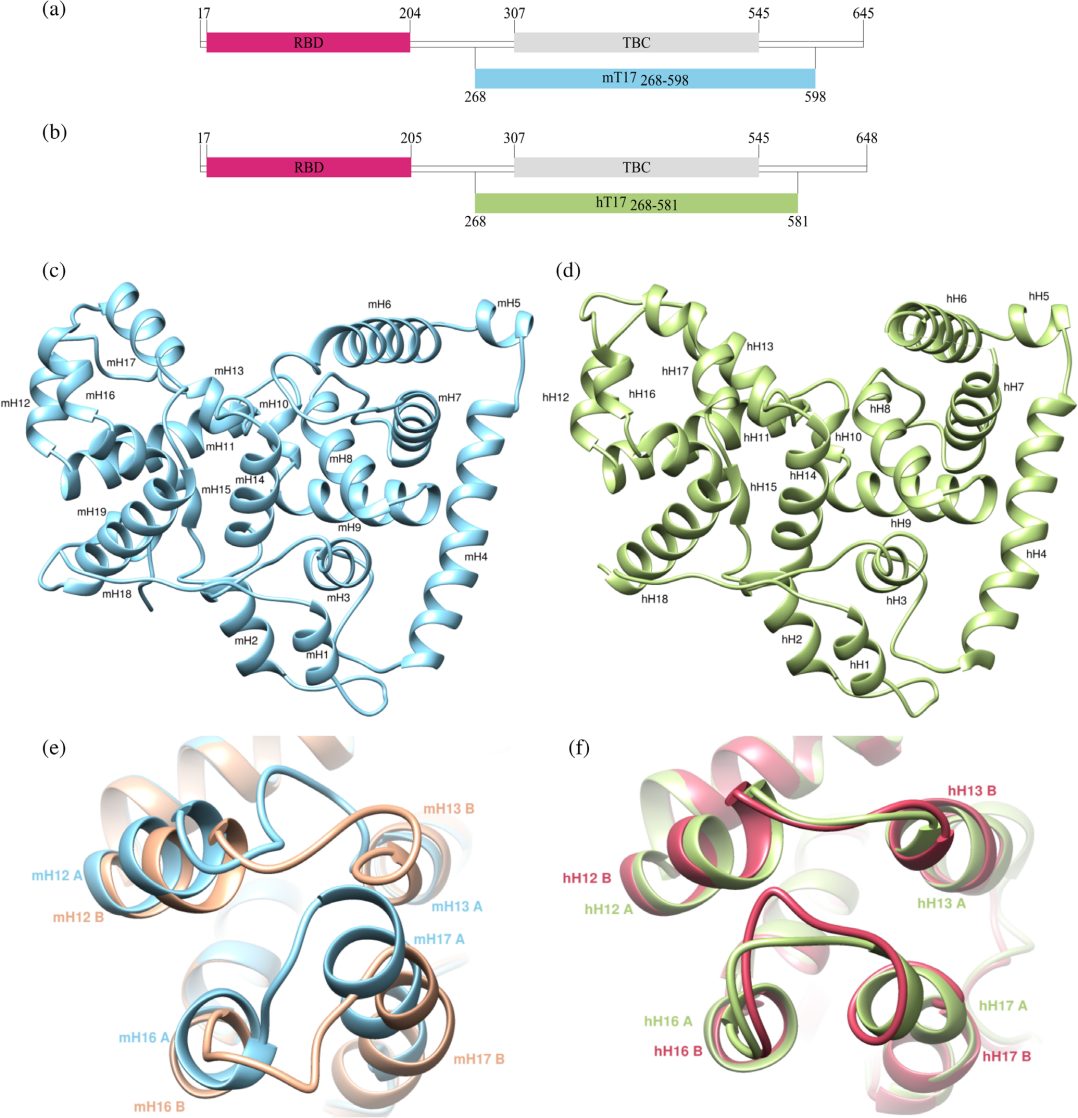
Overall structure of the TBC domain of TBC1D17. Schematic presenatation of murine (a) and human (b) TBC1D17 proteins with marked Rab‐binding (dark pink) and TBC (gray) domains. Fragments used for crystallization—mT17_268‐598_ (light blue) and hT17_268‐581_ (light green) are presented beneath the corresponding schemes. Solved structure of chains A of mT17_268‐598_ (c) with helices numbered from mH1 to mH19 and (d) hT17_268‐581_ with numbered helices from hH1 to hH18. (e) Superposition of chain A (blue) and B (light brown) of mT17_268‐598_ and (f) of chain A (light green) and B (light pink) of hT17_268‐581_, illustrating differences in the course of the main chain.

Full length murine and human TBC1D17 proteins contain 645 and 648 amino acids, respectively. As the TBC domain plays a crucial role in stimulating the activity of Rab proteins, our initial studies focused on this domain. According to the UniProt database (Bateman et al., 2025), the TBC domain constitutes a fragment spanning from 310 to 520 amino acids in both proteins. Based on the TBC domain structure of TBC1D15 (PDB ID: 5TUC) (Chen et al., 2017), showing 64% amino acid sequence identity to TBC1D17 and containing additional structural elements preceding the TBC domain, we designed a murine protein construct, covering 268 to 598 amino acid residues (mT17_268‐598_, Figure 1a) and an analogous human protein variant—hT17_268‐598_. Both proteins were efficiently produced in the bacterial expression system based on *Escherichia coli* BL21 Gold. However, because attempts to obtain hT17_268‐598_ crystals were unsuccessful, we designed a C‐terminally truncated variant spanning residues 268–581 (hT17_268‐581_, Figure 1b), based on the 5TUC structure. This construct was successfully purified and crystallized. The crystal of mT17_268‐598_ diffracted X‐rays to 2.2 Å and represented a space group *P*2_1_2_1_2 (Table 1). The analysis of X‐ray diffraction data obtained for hT17_268‐581_ protein (Table 1) indicated twinning of the crystal and the final resolution was established at 3.34 Å (*P*3_2_ space group).

**TABLE 1:** Data collection and refinement statistics.

Structure	mT17_268‐598_	hT17_268‐581_
Data collection		
Beamline	BioMAX, MAX IV, Lund	P13 PETRA III, EMBL, Hamburg
Wavelength (Å)	0.976	0.976
Temperature (K)	100	100
Space group	*P*2_1_2_1_2	*P3*_*2*_
Unit cell parameters		
*a*, *b*, *c* (Å)	94.24, 160.90, 41.90	97.98, 97.98, 82.28
*α*, *β*, *γ* (°)	90, 90, 90	90, 90, 120
Oscillation range (°)	0.2	0.2
Resolution (Å)	46.66–2.20 (2.33–2.20)	49.04–3.34 (3.63–3.34)^a^
Reflections collected/unique	110,744/32,171 (17,915/5,195)	92,299/8,626 (4,567/432)
Completeness (%)		
Spherical	96.7 (98.5)	67.5 (15.3)
Ellipsoidal	—	92.5 (73.3)
CC_1/2_	0.996 (0.648)	0.999 (0.745)
Multiplicity	3.4 (3.4)	10.7 (10.6)
*R*_merge_ (%)	11.4 (73.0)	13.8 (76.8)
*R*_pim_ (%)	—	4.5 (24.8)
<*I*/*σ*(*I*)>	8.0 (1.7)	13.5 (4.1)
Refinement		
*R*_free_ reflections	1636	1085
No. of atoms (non‐H)		
Protein	5327	4948
Ligands	0	0
Solvent	271	26
*R*_work_/*R*_free_ (%)	17.9/25.3	21.5/28.7
Mean ADP^b^ (Å^2^)	45.0	66.0
RMSD from ideal geometry		
Bond lengths (Å)	0.01	0.004
Bond angles (^o^)	1.97	1.18
Ramachandran statistics (%)		
Favored	97	88
Allowed	3	10
Outliers	0	2
PDB code	9SI6	9SI7

*Note*: Values in parentheses refer to the highest‐resolution shell.

^a^ Lowest cut‐off diffraction limit is 4.26 Å. Highest cut‐off diffraction limit is 3.34 Å.

^b^ Atomic displacement parameter.

Asymmetric units of both mT17_268‐598_ and hT17_268‐581_ are composed of two molecules. Due to a lack of an electron density map, in the murine structure residues 268–271 and 597–598 in chain A, as well as 268–273 and 596–598 in chain B, were not modeled. In the human structure, the electron density map for the N‐terminal residues 268–272 (chain A) and 268–273 (chain B), and for the loops spanning from 386 to 394 (chain A) and 389 to 392 (chain B) is not visible.

The full‐length murine and human TBC1D17 proteins share 89.3% sequence similarity, which increases to 93.9% within the fragment covered by the solved structure. Both structures superpose with RMSD of 1.3 Å (chains A)/1.1 Å (chains B). Three‐dimensional structures of TBC domains solved so far are described as V‐shaped (Rak et al., 2000). The same applies to the TBC domain of TBC1D17 proteins; however, due to the slightly different course of the helices within these domains, a heart‐like shape is more appropriate (Figures 1c,d and S1).

TBC domain of both murine and human TBC1D17 proteins is composed of helices and loops exclusively. The protein construct of mT17_268‐598_ (Figure 1c) is longer at the C‐terminus; therefore, the structure contains an additional H19 α‐helix, while the structure of hT17_268‐581_ (Figure 1d) presents 18 helices. As with other GAP proteins, in both structures two distinct subdomains can be distinguished: the first comprising helices H1–H10 and the second H11–H18 (human)/H19 (mouse).

There are differences between A and B chains in both human and murine structures. Superposition of chains A and B in human structure yields RMSD of 0.8 Å, and in murine—0.9 Å. While the first subdomain is virtually identical in both structures, the greatest differences between chains were observed in the second subdomain, especially comparing fragments of H12, H13, H16, H17 and the loops between them. Those differences are more noticeable in the murine protein (Figure 1e). Although the length of mH12 is identical in both chains, the divergence becomes evident starting from the residue Asp_479_. The following loops present variable conformations and in the furthest point, constituting Gly_487_, the distance between Cα of this residue in A and B chains is about 11 Å. In turn, the beginning of the helix mH13 in chain A constitutes Phe_493_, while in chain B it starts three amino acids earlier. In the case of mH16, the situation is opposite—in chain B, the helix is three amino acids shorter than the corresponding helix in chain A, and these residues create a loop. Even though helix mH17 starts from the same Ser_548_ residue in both chains, its beginning in the B chain is located above 4 Å away from the A chain after superposition. Moreover, mH17 B is four residues longer than mH17 A and the first helix turn is narrower than the remaining ones. The above‐mentioned differences between A and B chains in murine structure arise probably from close contacts with other symmetrical macromolecules, due to packing of the protein molecules in the crystal. In chain A, mH12 and mH13 are located near A′ molecule, while mH16 and mH17 are adjacent to the B′ chain of the symmetrical molecule. In the case of the B chain, mH12, mH13, mH16 and mH17 are located nearby the B′ chain and additionally, mH16 and mH17 are also located at the surface of interaction with chain A′. What is more, the aforementioned fragments are the most mobile parts, as indicated by high B‐factors and lack of well‐defined electron density map.

The differences in the human structure are much more subtle (Figure 1f) compared with those observed in the murine protein. The lengths of hH12 and the following loop, as well as hH16, are identical in both chains. The most significant differences occur in the region spanning the loop connecting hH16 and hH17 helices and in hH17 itself. In chain A, hH17 is slightly curved and starts from Asn_549_, while in chain B it starts at Asn_566_, with the preceding residues forming a longer loop.

### 2.2 Oligomeric form of TBC domains of TBC1D17

**FIGURE 2:**
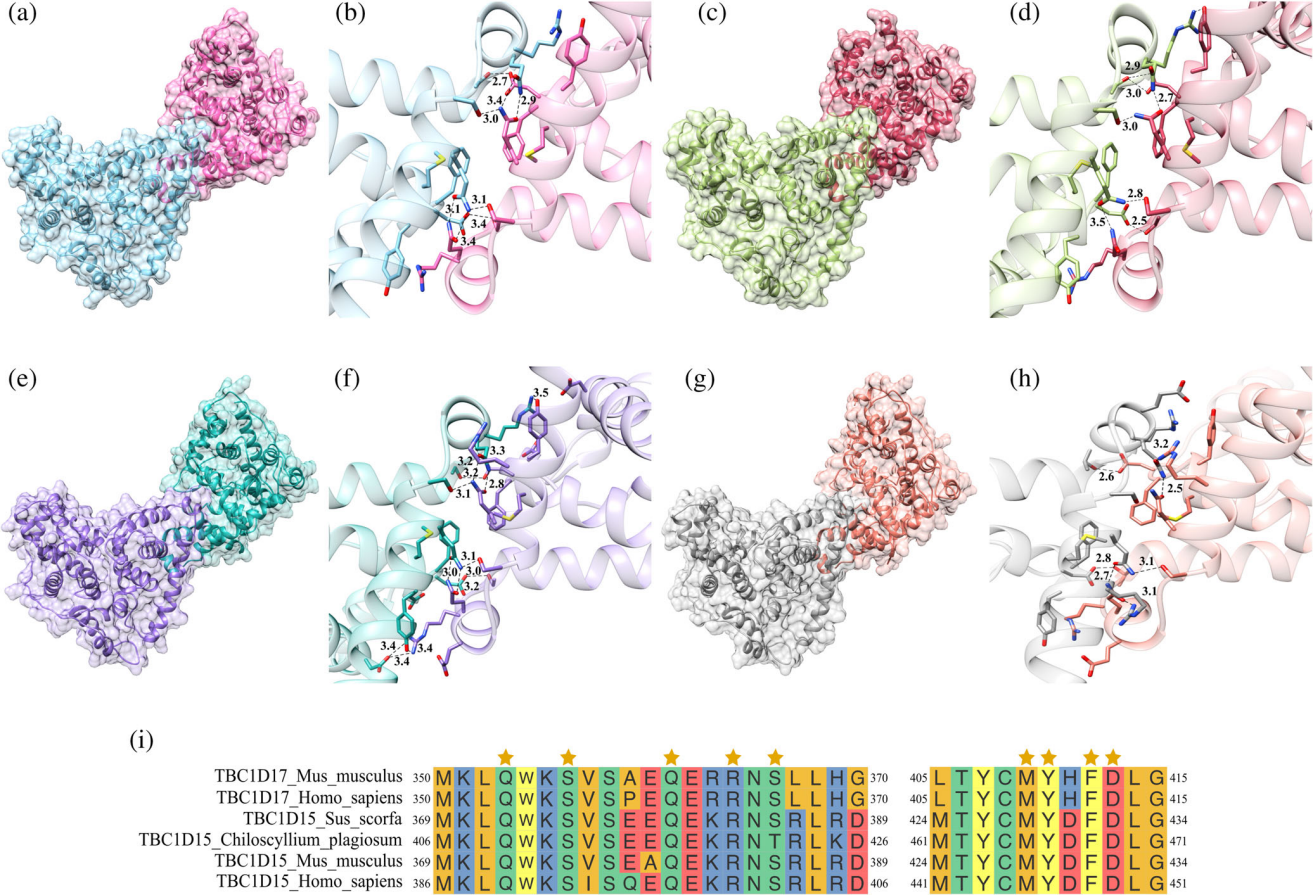
Oligomeric form of the TBC domain of TBC1D17 and TBC1D15. Spatial arrangement of chains A and B and the interaction surface of (a, b) mT17_268‐598_, (c, d) hT17_268‐581_, and chains A and B′ of (e, f) TBC domain of *Sus scorfa* TBC1D15 (PDB ID: 5TUC) and (g, h) TBC domain of *Chiloscyllium plagiosum* TBC1D15 (PDB ID: 5TUB). The figures show the distances measured in angstroms (Å) between the side chains of amino acids detected by PISA. In the case of hT17_268‐581_ where PISA did not detect any interactions, the side chains of amino acids analogous to mT17_268‐598_ are presented. (i) Protein sequence alignment of TBC1D17 and TBC1D15 with known structures and additional mice and human sequences of TBC1D15 with residues involved in interchain interactions indicated with stars.

Analysis of interacting surfaces within mT17_268‐598_ with the PISA server revealed that this protein can create a dimer; however, its predicted stability is low. The dimer is created by chains A and B constituting an asymmetric unit of the unit cell (Figure 2a). Residues located on mH4, mH5, mH6, mH7, and the N‐terminal fragment of the loop located behind helix mH7 (Figure 2b) participate directly in the creation of hydrogen bonds. Among 21 detected hydrogen bonds, eight have been identified between side chains of amino acids. Interestingly, no interactions suggesting formation of the dimer in the crystal were detected by PISA for hT17_268‐581_. However, analyzing the arrangement of chains A and B in space relative to each other, a similar spatial conformation can be noticed (Figure 2c). Additionally, the same amino acids can be found at the interaction surface, suggesting the potential formation of a dimer (Figure 2d). This observation is consistent with gel filtration results, as both mT17_268‐598_ and hT17_268‐581_ are eluted from the column in the volumes corresponding to their dimeric form. Due to the discrepancy between our observations and PISA results for hT17_268‐581_, we performed an additional analytical size‐exclusion chromatography (SEC) (Figure S2a) that confirmed hT17_268‐581_ eluting at a volume corresponding to ~75 kDa relative to globular standards (conalbumin is 75 kDa). Given that the monomer mass would be ~36 kDa, this elution profile strongly suggests the presence of a dimer in solution. Additionally, we performed dynamic light scattering measurements (Figure S2b), and the obtained hydrodynamic diameter of hT17_268‐581_ is 8.4 nm, indicating that the protein cannot represent the monomeric state in solution. The value seems to be high even for the dimer. However, considering the elongated shape of the molecule, we also measured the distance between the most distant rigid molecule fragments in the hT17_268‐581_ structure, and the obtained value of 8.7 nm correlates well with the measured hydrodynamic diameter. The reason of inability to detect intermolecular interactions by PISA in hT17_268‐581_ may be protein crystal imperfections arising from twinning and causing too close contacts between molecules (clashes) that hinder PISA analysis.

It is worth noting that similar interactions as in mT17_268‐598_ have been detected by PISA between A and B′ molecules of the TBC domain of TBC1D15 protein in *Sus scorfa* (PDB ID: 5TUC, Figure 2e,f) and *Chiloscyllium plagiosum* (PDB ID: 5TUB, Figure 2g,h) structures. In both TBC1D15 structures, the number of hydrogen bonds at the interaction surface is higher than at the murine TBC1D17 structure and amounts to 29 and 25 hydrogen bonds respectively, among which 18 (Figure 2f) and 12 (Figure 2h) are between side chains. Furthermore, in the *Sus scorfa* structure, six salt bridges are formed between A and B′ chains. However, the larger number of interactions in TBC1D15 structures does not improve the stability of the homodimer according to PISA. Protein sequence alignment of TBC1D17 and TBC1D15 from different organisms, for which the structures were solved, revealed that the residues involved in interchain interactions are identical (Figure 2i). Moreover, in both mouse and human TBC1D15, for which structures are not yet available, the same amino acids are present on the corresponding aligned positions.

### 2.3 Analysis of the TBC domain conservation of TBC1D17

**FIGURE 3:**
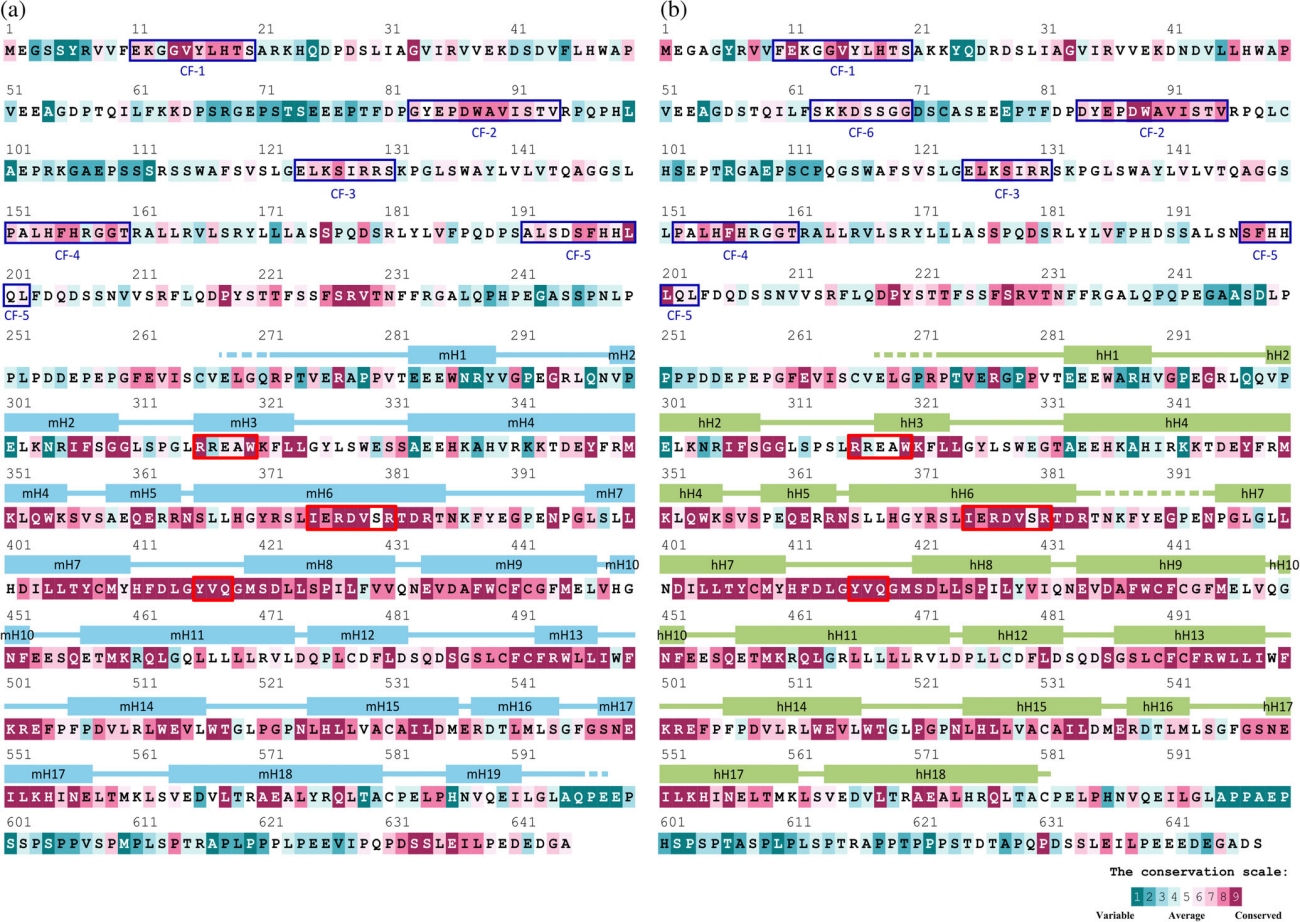
Conservation of the TBC1D17 amino acid sequence. Conservation of amino acids in (a) murine and (b) human TBC1D17 proteins across homologous proteins. Blue or green boxes above the sequences represent secondary structure of the proteins. mH1–mH19 marks present helices in murine protein, hH1–hH18 helices in human protein, while remaining parts are unstructured loops. Red rectangles indicate sequential motifs crucial for GAP activity and structural integrity of TBC1D17 protein. Conserved fragments within RBD (CF 1–6) are highlighted by the navy blue rectangles.

The TBC domain is highly conserved among homologous proteins originating from different species (Fukuda, 2011). The same applies to the TBC domain of the TBC1D17 protein, as analysis of mouse and human homologs revealed a strong conservation of amino acids within this region (Figure 3a,b). The highest variability has been observed in helices H1 and N‐terminus of H2 preceding the actual TBC domain and in the N‐terminal fragment of a H4 helix (residues 333–342), while the remaining regions of the TBC domains are highly conserved among TBC1D17 and TBC1D15 homologs. The fragments with the highest scores in the conservation scale involve helices H6 and a long segment starting from the C‐terminus of helices H7 and ending on helices H9. In these fragments, two highly conserved motifs can be distinguished—I_375_xxD_378_xxR_381_ and Y_416_xQ_418_ (Pan et al., 2006), which support the catalytic activity of Rabs with which they interact. Another conserved motif, R_316_xxxW_320_, which serves a structural function, is located in helix H3.

Conservation of amino acids in a given position in TBC1D17 proteins is less visible when analyzing sequences of TBC domain containing GAPs from a single species—*Mus musculus* (Figure S3a) or *Homo sapiens* (Figure S3b). However, it is still noticeable that the H7‐H9 segment constituting the core of the TBC domain is more conserved than the other part of this domain. The highest conservation scores were assigned to the aforementioned catalytic and structural motifs.

### 2.4 Modeling of the full‐length TBC1D17 structure and predictive structural analysis of its Rab‐binding domain

**FIGURE 4:**
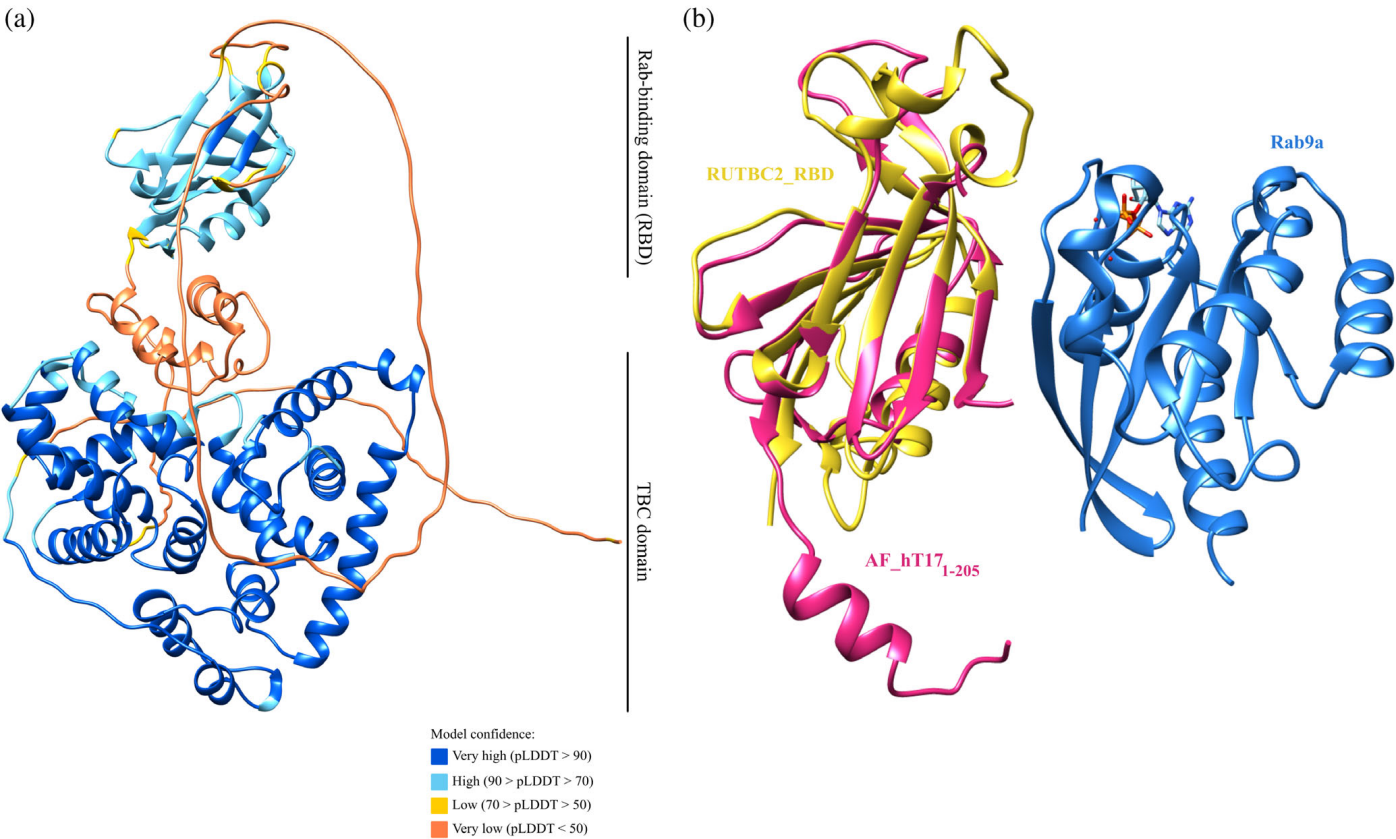
Predicted model of the full‐length TBC1D17. (a) Model of full‐length TBC1D17 protein predicted by AlphaFold 2 with two distinct domains—N‐terminal Rab‐binding domain (RBD) and C‐terminal TBC domain. Structure is colored based on confidence score of the model. (b) Superposition of the complex between RUTBC2_RBD (yellow) and Rab9a (blue) (PDB ID: 4QXA) with human TBC1D17 RBD model—AF_hT17_RBD (dark pink) generated by AlphaFold 2. For improved clarity of the figure, fragment of AF_hT17_RBD spanning from 54 to 113 is hidden.

Analysis of the TBC1D17 sequences using the InterPro database (Blum et al., 2025) revealed the presence of N‐terminal Rab‐binding domain (RBD), spanning residues 7–204 in mouse and 7–205 in human, with 85.8% sequence identity between them. Notably, this domain was not annotated in the UniProt database. To verify the role of this domain, we have prepared two additional human TBC1D17 constructs—hT17_1‐205_ (containing 1–205 amino acid residues) and hT17_1‐581_ (covering the fragment from 1 to 581 amino acids and both RBD and TBC domains). Unfortunately, we could not crystallize any of them. For this reason, a model of a full‐length human TBC1D17 protein generated by AlphaFold 2 (AlphaFold ID: AF‐Q9HA65‐F1, referred to in the manuscript as AF_hT17) (Jumper et al., 2021; Varadi et al., 2022, 2024) was used for bioinformatic analyses (Figure 4a).

The TBC domain of the AF_hT17 model superimposed with hT17_268‐581_ with RMSD of 1.3 Å, which is consistent with very high model confidence on this fragment (Figure 4a). According to the RBD model of AF_hT17, it is built from 10 β‐strands and one α‐helix, characterized by a high model confidence score. Simultaneously, predicted loops spanning residues 20–28 and 54–115, which connect the aforementioned secondary structures, have a very low model confidence score. The only structure of RBD available in the PDB is a complex between mice RUTBC2_RBD and Rab9a (PDB ID: 4QXA) (Zhang et al., 2014). Sequence identity between mice RUTBC2_RBD (also known as SGSM1) and hT17_1‐205_ calculated by Clustal Omega (Madeira et al., 2024) is 22.5%; however, the general fold of predicted structure is similar to RUTBC2_RBD, and superposition of the AF_hT17_RBD and RUTBC2_RBD gives RMSD of 2.1 Å (Figure 4b). In turn, sequence identity between murine Rab9a and human Rab5a is 33.7% and superpose with RMSD of 1.4 Å (PDB ID: 1N6H) (Zhu et al., 2003). Rab proteins interact with their partners mainly through switch I, interswitch and switch II regions. In the Rab9a‐RUTBC2 structure, all residues of Rab9a involved in interactions are located within these fragments (Phe_37_, Ile_40_, Gly_41_, Val_42_, Glu_43_, Phe_44_, Trp_61_, Arg_68_, Phe_69_, Leu_72_, Phe_76_). Five out of these 11 residues of Rab9a are conserved in Rab5a (Ile_53_, Gly_54_, Phe_57_, Trp_74_, Arg_81_). Hence, we decided to check whether the interaction interface of TBC1D17_RBD‐Rab5a could be similar to the one of the RUTBC2_RBD‐Rab9a complex. The interaction interface of RUTBC2_RBD with Rab9a constitutes the following residues: Leu_256_, Lys_260_, Asn_261_, Asn_262_, Tyr_277_, Asn_294_, Met_297_, Asn_298_ and Gln_361_ of RUTBC2 (Zhang et al., 2014). When superimposed, these residues correspond to Val_8_, Lys_12_, Gly_13_, Gly_14_, Val_34_, Val_51_, Ala_54_, Gly_55_, Ala_163_ of AF_hT17_RBD. Residues 54 and 55 are predicted to lie at the beginning of the long, low confidence loop according to Alpha Fold model and do not overlap with the corresponding residues in RUTBC2_RBD. As most of these hT17_1‐205_ residues are completely deprived or having a very short side chain, it is unlikely that they are responsible for direct interaction with Rab5a protein. This assumption may also be supported by the fact that the analyzed fragment of hT17_1‐205_ does not contain Trp, Arg or Tyr residues, which are favorable to create PPIs between different proteins (Moreira et al., 2007). It is worth emphasizing that presented bioinformatic analyses should be considered as predictive. It is also possible that the Rab5a–TBC1D17 interaction is mediated by the mentioned loops of RBD which exhibit low pLDDT value in the AlphaFold model, which is known as a good disorder predictor (Vander Meersche et al., 2025).

### 2.5 RBD involvement in the interaction with Rab5a

**FIGURE 5:**
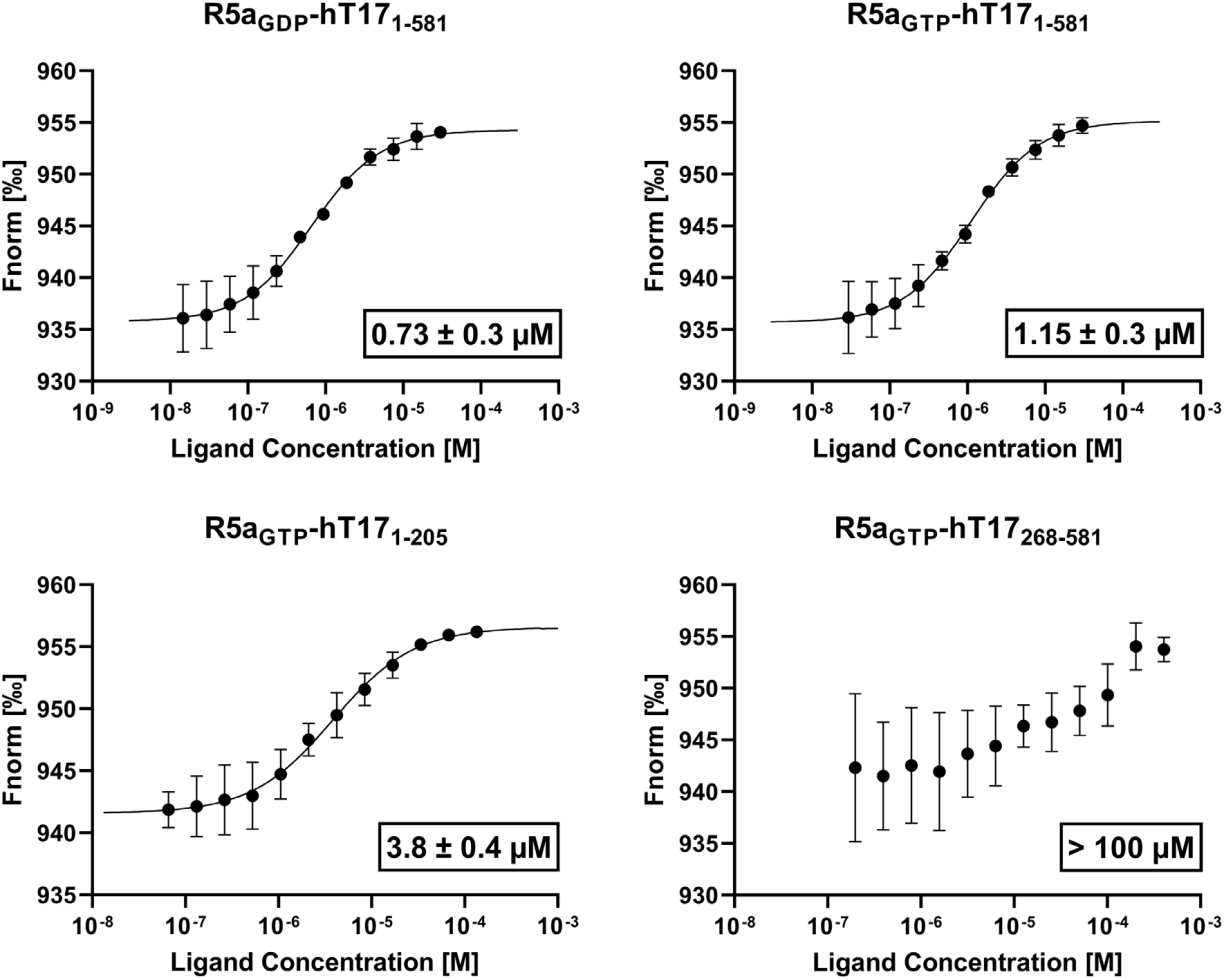
The results of the MST measurements. The MST curves representing the interactions between Rab5a protein and variants of TBC1D17: HT17_1‐205_, hT17_268‐581_, and hT17_1581_. The values shown below each curve present *K*_d_ ± SD.

To verify whether RBD is necessary for Rab5a‐TBC1D17 complex formation, hT17_1‐205_, hT17_268‐581_, and hT17_1‐581_ and quantify the strength of the interaction, Monolith X (NanoTemper, Germany) was used. We determined dissociation constants using Microscale Thermophoresis (MST). The MST curves representing the interactions between Rab5a protein and variants of TBC1D17 were presented in Figure 5. The curves for all individual repetitions can be found in Figure S4. The strongest interaction was detected for Rab5a bound with GDP and hT17_1‐581_ (*K*_d_ of 0.73 ± 0.3 μM). The interaction of Rab5a in complex with GTP and hT17_1‐581_ was only slightly weaker, with *K*_d_ of 1.15 ± 0.3 μM. MST analysis confirmed interaction between Rab5a and hT17_1‐205_ with *K*_d_ of 3.8 ± 0.4 μM, which supports the assumption that RBD is important for Rab5a binding. With this assay, we were able to detect the interaction between Rab5a loaded with GTP and the TBC domain (hT17_268‐581_), but the binding affinity was significantly lower (*K*_d_ above 100 μM), which additionally highlights the significance of the RBD domain in mediating the interaction with Rab5a.

### 2.6 Analysis of the RBD conservation and phylogenetic tree of TBC domain‐containing GAPs

As mentioned earlier, the attempts to crystallize RBD of TBC1D17 alone or in complex with Rab5a failed. However, as we observed the formation of the complex between RBD and Rab5a, we decided to identify which residues can be directly involved in PPI. Thus, we performed analysis of amino acid conservation, in search of residues with the highest score, which could suggest their engagement in creation of the complex. The sequence of TBC1D17 RBD, which spans 7–204 (mouse) or 7–205 (human) amino acids, is much more variable across homologous proteins compared to the TBC domain. There are only five (mouse) and seven (human) residues with the highest conservation score (Figure 3a,b). Two of them, Val_15_ and Gly_33_, are conserved in a given position in both mouse and human TBC1D17; however, their role is unknown. Additionally, five conserved regions can be distinguished in mice and human domains: 11–20 (mouse)/10–20 (human) (CF‐1), 83–94 (mouse)/84–95 (human) (CF‐2), 124–131 (mouse)/125–131 (human) (CF‐3), 151–160 (mouse)/152–161 (human) (CF‐4) and 192–202 (mouse)/197–203 (human) (CF‐5). Human RBD contains an additional conserved fragment from 63 to 70 residue (CF‐6); however, its score is not high.

Conservation scores decreased when analysis was limited to GAP proteins originating from a single organism (Figure S3). This result is not surprising, as besides TBC1D17, only three other proteins in TBC domain containing GAPs have the annotated RBD: TBC1D15, SGSM1 and SGSM2; however, the two latter ones are sequentially distant from the sequence of TBC1D17 and TBC1D15. Only one segment (residues 12–21) in the mouse sequence overlaps with the CF‐1 identified in homologous TBC1D17 proteins (Figure S3a). In human TBC domain‐containing GAPs, conservation of previously mentioned segments is lower; however, another fragment spanning from 142 to 155 with high score can be identified (Figure S3b). Finally, residues with the highest conservation scores are Pro_82_ and Ser_196_ in mice and Pro_50_, Pro_110_ and Pro_178_.

RBD and TBC domains are connected through a linker spanning from 205 (mouse)/206 (human) to 306 residues. Considering homologous proteins (Figure 3a,b), two conserved segments are visible in TBC1D17: 217–231 (mouse)/218–232 (human) and 260–264 (mouse)/258–264 (human). According to Vaibhava et al. residues 218–309 are necessary for interaction with optineurin (Vaibhava et al., 2012). However, research conducted by Zhang et al. indicates that a construct starting from residue 274 is already sufficient for this interaction (Zhang et al., 2024). Although structural data for the fragment comprising residues 218–309 are not available, the conservation of several residues in this fragment may indicate their role in interactions with other proteins.

**FIGURE 6:**
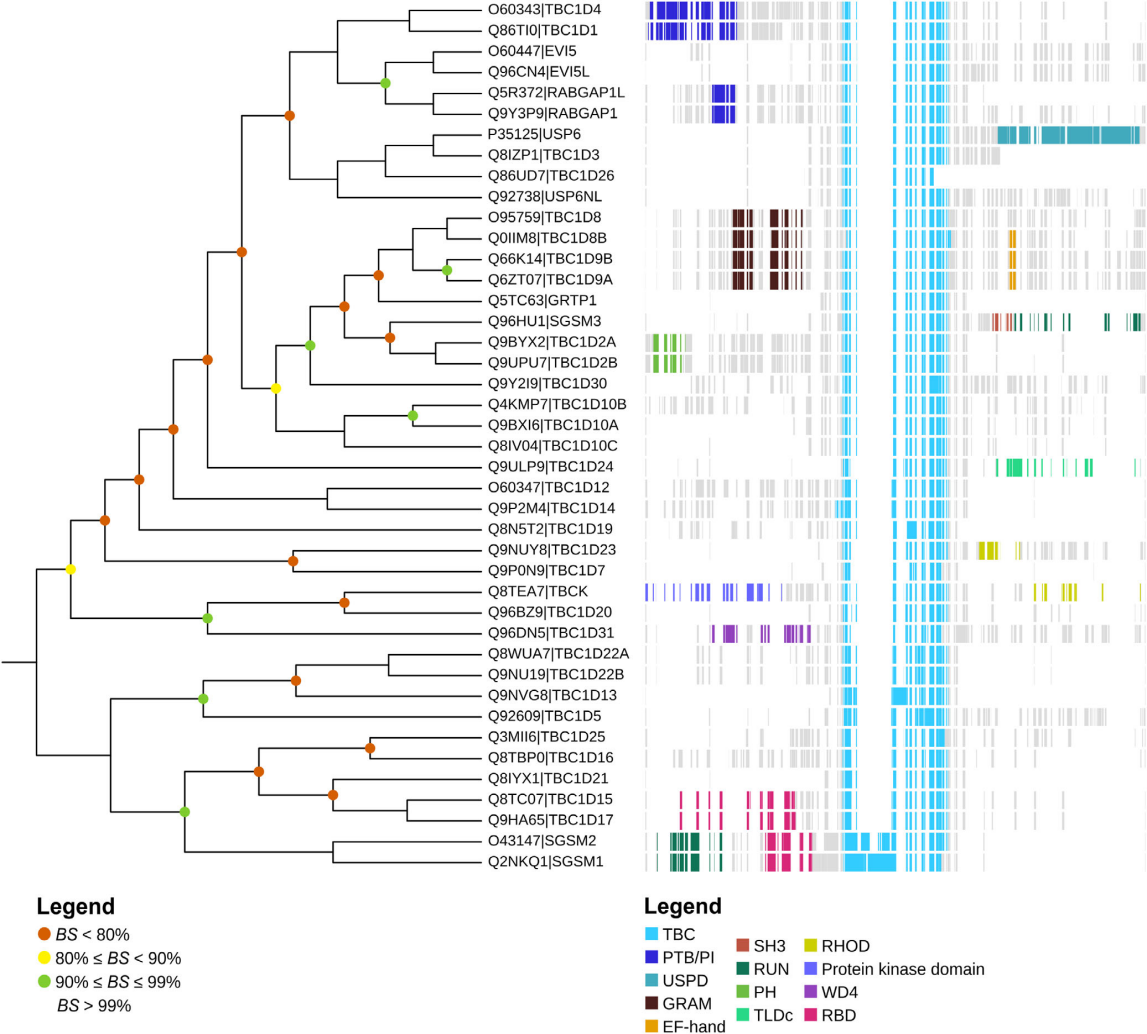
Phylogenetic tree of TBC domain‐containing GAPs originating from *Homo sapiens*. Bootstrap values (BS) indicate confidence in clustering of proteins into the branch. EF‐hand helix, loop‐helix structural domain; GRAM, glucosyltransferases, Rab‐like GTPase activators and myotubularins domain; PH, Pleckstrin homology domain; PTB/PI, phosphotyrosine binding/phosphotyrosine interacting domain; RBD, Rab‐binding domain; RHOD, rhodanese homology domain; RUN, RPIP8, UNC‐14 and NESCA domain; SH3, Src homology 3 domain; TBC, Tre‐2/Bub2/Cdc16 domain; TLDc, TBC and LysM domain‐containing; USPD, ubiquitin specific protease domain; WD4, tryptophan‐aspartic acid repeat/beta‐transducin repeat.

As the structure of TBC1D17 RBD could not have been resolved experimentally, the best course of action is to model and compare it with a highly similar protein with a known binding mode. The phylogenetic tree of human TBC domain‐containing GAPs was generated using IQ‐TREE software (Figure 6). Many of the analyzed GAPs contain additional domains like RUN, PTB; however, among the closely related TBC1D17 homologs, the RBD was identified only in TBC1D15 (clustered together with TBC1D17), SGSM1 (RUTBC2), and SGSM2 (RUTBC1), belonging to the same parental clad. Although TBC1D21, TBC1D16, and TBC1D25 are closer homologs of TBC1D17 than SGSM1 and SGSM2, these GAPs do not contain any additional domains. In contrast, SGSM1 and SGSM2 differ significantly from all other GAPs analyzed in the tree, as these proteins have the N‐terminal RUN domain, prior RBD, and their TBC domain also differs significantly from other GAPs, including TBC1D17. In summary, the presence of the RBD in TBC1D17 and TBC1D15 is a feature that makes these GAPs unique and the experimental structure of RBD, which would be highly similar to the one in TBC1D17, is currently not available. Hence, the closest homolog with experimentally determined RBD structure is the one analyzed in previous sections RUTBC2 (SGSM1).

## 3 DISCUSSION

Gyp1 (PDB ID: 1FKM), a yeast homolog of human TBC1D22B solved in 2000, is the first structurally characterized GAP, which is able to interact with Rab proteins (Rak et al., 2000). Six years later the structure of the complex between Gyp1 and Rab33 (PDB ID: 2G77) was published (Pan et al., 2006). Both Gyp1 structures adopt a V‐shaped conformation and superpose with hT17_268‐581_ and mT17_268‐598_ with RMSD of about 2 Å, with a sequence identity of 24% (Table 2). A similar three‐dimensional structure was observed for human TBC1D22A and TBC1D22B proteins (PDB ID: 2QFZ and 6D0S). The main differences within the TBC domain encompass the number and length of α‐helices. While Gyp1, TBC1D22A, and TBC1D22B are built from 16 α‐helices, TBC1D17 protein contains 2 (human) or 3 (mice) additional helices—H5, H10, and mH19. In both Gyp1 structures, there is a fragment spanning from 510 to 569 (PDB ID: 1FKM) or 511 to 567 (PDB ID: 2G77) residues that cannot be tracked in electron density maps. It was predicted that additional helices can be formed in this fragment according to Jpred (Rak et al., 2000). Moreover, Gyp1, TBC1D22A, and TBC1D22B contain a long loop located between the 7th and 8th helix (corresponding to the fragment between H8 and H9 in hT17_268‐581_ and mT17_268‐598_), which is absent in analyzed TBC1D17 structures due to the lack of electron density in this region (Pan et al., 2006; Rak et al., 2000). TBC1D17 exhibits the highest structural similarity (Table 2) and sequence identity with TBC1D15 proteins from shark and wild boar (PDB ID: 5TUB, 5TUC) (Chen et al., 2017). In shark TBC1D15 structure, 20 helices can be distinguished, while in wild boar TBC1D15 the first α1′ helix is not formed, thus it is built from 19 helices. In contrast to Gyp1, TBC1D15 and TBC1D17 contain helix H5 (α4′ in Chen et al. (2017) study), while helix corresponding to H10 in TBC1D17 constitutes fragment of α8 helix in TBC1D15 structures.

**TABLE 2:** Comparison of the hT17_268‐581_ and mT17_268‐598_ structures with the proteins with the highest structural similarity according to PDBeFold (Krissinel & Henrick, 2007).

Protein	Organism	PDB ID	RMSD^a^ [Å]	Sequence identity^b^ [%]	RMSD^c^ [Å]	Sequence dentity^d^ [%]	References
TBC1D15	*Sus scorfa*	5TUC	1.35	63	0.8	64	Chen et al. (2017)
TBC1D15	*Shark (Squalomorphi)*	5TUB	1.32	59	0.91	59	
TBC1D22A	*Homo sapiens*	2QFZ	1.86	25	2.06	24	NA
Gyp1 + Rab33	*Saccharomyces cerevisiae* *Mus musculus*	2G77	1.92	24	2.07	24	Pan et al. (2006)
TBC1D22B	*Homo sapiens*	6D0S	1.92	24	2.29	21	NA
Gyp1p	*Saccharomyces cerevisiae*	1FKM	2.00	24	2.05	24	Rak et al. (2000)
TBC1D20 + Rab1b	*Homo sapiens*	4HLQ	2.01	21	2.12	22	Gavriljuk et al. (2012)
TBC1D20	*Homo sapiens*	4HL4	2.05	21	2.02	22	
RabGAP	*Chlamydomonas reinhardtii*	4P17	2.22	24	3.04	22	Bhogaraju and Lorentzen (2014)
RABGAP1	*Homo sapiens*	4NC6	2.23	20	2.57	20	NA
TBC1D14	*Homo sapiens*	2QQ8	2.68	20	2.37	20	NA
TBC1D7 + TSC1	*Homo sapiens*	5EJC	2.83	17	2.67	17	Qin et al. (2016)

*Note*: NA, reference not available.

^a^ RMSD value obtained after superposition of Cα atoms of hT17_268‐581_ chain A.

^b^ Sequence identity on aligned fragment relative to hT17_268‐581_.

^c^ RMSD value obtained after superposition of Cα atoms of mT17_268‐598_ chain A.

^d^ Sequence identity on aligned fragment relative to mT17_268‐598_.

It is of note that all discussed structures of homologs represent TBC domains of GAP proteins. While many studies have focused on this domain, some GAPs contain additional domains. According to our analyses, RBD of TBC1D17 is crucial for its interaction with Rab5a, which is supported by lower binding affinity of Rab5a towards the TBC domain in comparison to RBD or the RBD and TBC‐containing protein. Previously, the interactions between TBC1D17 and Rab5a were analyzed by Rao et al. using a pull‐down assay. According to their studies, the inactive GDP‐bound Rab5a was preferred by TBC1D17 over the active, GTP‐bound Rab5a (Rao et al., 2021). Similar results were obtained for TBC1D4 as the binding affinity towards Rab2a loaded with GDP was much higher than loaded with GTP (Tian et al., 2024). Interestingly, there are some examples of GAP proteins interacting with both GTP‐ and GDP‐loaded forms with similar efficiency. It includes TBC1D2B, TBC1D11, and TBC1D6 proteins interacting with Rab22a, Rab36, and Rab26 respectively (Kanno et al., 2010; Wei et al., 2019). Finally, an ability to bind GDP‐bound Rab was also observed for SGSM2 (RUTBC1) and Rab9a, however, with much lower efficiency compared to GTP‐loaded Rab9a (Nottingham et al., 2011). These observations align with our results, as the affinity of hT17_1‐581_ was slightly higher for the GDP‐bound Rab5a than for the GTP‐bound Rab5a. However, our findings indicate a different pattern of interactions between TBC1D17 and Rab5a, compared to the results obtained by Rao et al. (2021). The results of their pull‐down assays showed the interaction between the TBC domain of TBC1D17, while the TBC1D17 N‐terminal RBD‐containing fragment and the C‐terminal fragment comprising the proline‐rich motif did not pull down Rab5a. In turn, in our MST analysis we identified a strong interaction between TBC1D17‐RBD and Rab5a, while interaction with hT17_268‐581_ occurs, but with lower affinity than when RBD is present. All the discrepancies may stem from differences in the construct design. In the studies of Rao et al. (2021), the murine TBC domain covered fragment from 307 to 545. In our case, protein constructs span 268–581 (human) or 268–598 (mouse) residues. The design guarantees full coverage of the TBC domain, with additional helices (mH1, mH2, and mH15–mH19 as well as hH1, hH2, and hH15–hH18) surrounding the actual domain, which were omitted in the Rao et al. (2021) construct. Our design is supported by AlphaFold prediction, as in the mouse and human TBC1D17 models, the N‐terminal domain is clearly separated from the C‐terminal domain, which gave us certainty to overproduce a stable domain. Moreover, such a design of the TBC domain‐containing fragment leads to the formation of a dimeric form of this protein, which was confirmed by SEC, DLS and crystal contact analysis of our structures and other homologs. The dimer interface in mouse and human TBC1D17 includes residues located in helices H4, H5, H6, H7 and the loop following H7. They are situated in the immediate neighborhood of highly conserved motifs IxxDxxR and YxQ, involved in direct interaction with GTP. This may decrease the affinity of Rab5a to the TBC domain in hT17_268‐581_. Dimerization of TBC domain‐containing GAPs has been observed in the case of EVI5 and TBC1D4 (Faitar et al., 2005; Woo et al., 2017). In these proteins, coiled‐coil motifs located on the C‐terminus are responsible for interactions between monomers. However, there is a report where a truncated TBC1D4 fragment comprising residues 1–363, which corresponds to PTB domains according to our analysis, can create a dimer with full‐length TBC1D4 (Dash et al., 2009). It is worth noting that dimerization of TBC1D4 (containing TBC domain and coiled‐coil fragment) did not affect its ability to stimulate GTP hydrolysis in vitro by Rab14, in comparison to monomeric TBC1D4 construct containing only the TBC domain (Woo et al., 2017). The TBC1D17 protein is deprived of both coiled‐coil fragment and PTB domain, and in contrary to Woo et al. (2017), we have observed differences in the ability to recognize Rab5a loaded with GTP or GDP. Finally, homodimerization of TBC1D17 protein has been observed by Yamano et al. (2014), which agrees with our observations. TBC1D15 and TBC1D17 mediate proper autophagic encapsulation of mitochondria and as suggested by the authors of the study the dimerization may increase their affinity for their protein partners by multivalent binding (Yamano et al., 2014). Interestingly, they also confirmed a heterodimer formation between TBC1D17 and TBC1D15. We analyzed sequences and crystal structures of hT17_268‐581_, mT17_268‐598_, and TBC1D15 proteins and revealed the presence of the same amino acids on the interaction surface. Thus, we hypothesize that interactions between TBC1D17 and TBC1D15 occur similarly to those observed in the presented structures. Heterodimers between GAPs have also been detected for TBC1D1 and TBC1D4 and this complex is likely formed through PTB domains (Hatakeyama et al., 2019).

**FIGURE 7:**
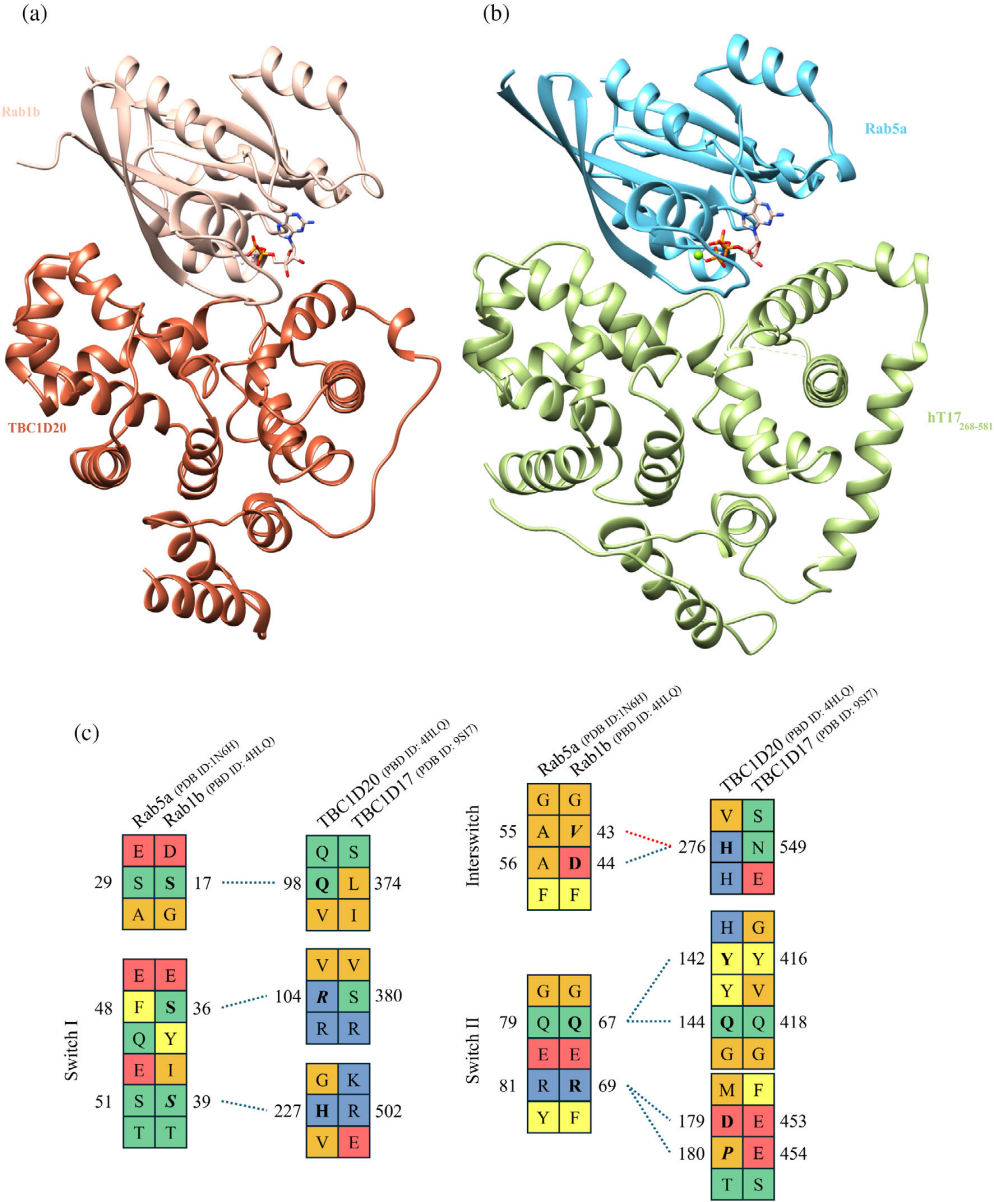
Comparison of interaction between the Rab1b‐TBC1D20 complex (PDB ID: 4HLQ) with potential complex of Rab5a (PDB ID: 1N6H) and TBC1D17 (this study, PDB ID: 9SI7). (a) The structure of the Rab1b‐TBC1D20 complex. (b) Potential complex of Rab5a and TBC1D17, obtained by superposition of the structures on the Rab1b‐TBC1D20 complex. (c) Comparison of the interacting and potentially interacting residues of Rab1b and Rab5a, as well as TBC1D20 and TBC1D17. Blue, dashed lines present hydrogen bonds and red, dashed lines present salt bridges between Rab1b and TBC1D20 detected by PDBSum (Laskowski, 2022). Residues directly involved in interactions are bolded and the ones interacting only via its main chain atoms are in italic. Residues colored by chemical character of its side chain (red—acidic, blue—basic, green—hydrophilic, orange—hydrophobic, yellow—aromatic).

The interruption of the interaction between TBC1D17 and Rab5a could have a therapeutic effect in treatment of diabetes, as the complex of these proteins is required for GLUT receptor translocation inhibition. To map potential interaction surfaces between the TBC domain of TBC1D17 and Rab5a, we compared the known structure of the TBC1D20‐Rab1b complex (PDB ID: 4HLQ) (Gavriljuk et al., 2012) (Figure 7a) with the potential structure of the hT17_268‐581_‐Rab5a complex (Figure 7b), obtained by superposition of the hT17_268‐581_ structure on TBC1D20 and the Rab5a structure (PDB ID: 1N6H) (Zhu et al., 2003) on Rab1a. Although both TBC1D17/TBC1D20 and Rab1b/Rab5a protein pairs are very similar, an analysis of primary and tertiary structures reveals key differences that prevent the exact application of TBC1D20‐Rab1b binding mode to TBC1D17‐Rab5a complex. The sequences of the proteins were aligned based on the tertiary structure superposition (Figure 7c). For the binding mode to remain conserved between homologous pairs, the substitution in one protein should be accommodated by a compensatory change in the other. Mapping the known TBC1D20‐Rab1b interaction sites onto the sequences of TBC1D17‐Rab5a pair reveals uncompensated differences across all the corresponding regions in either or both binding partners. For the Rabs, the switch II region remains nearly identical; however, the variations occur in the regions preceding switch I, within switch I, and in the interswitch regions. The first notable difference occurs outside the switch regions. The polar Q_98_ of TBC1D20 is substituted by a hydrophobic L_374_ in TBC1D17, even though the corresponding residue in Rab5a remains unchanged (S_17_/S_29_). Within the switch I region, the Rab1b _36_SYIS_39_ is replaced by _48_FQES_51_ in Rab5a and S_36_ important for the interaction in Rab1b is replaced by F_48_ in Rab5a. The most interesting change can be found in the interswitch region where the _42_GVDF_45_ of Rab1b is changed to more hydrophobic _54_GAAF_57_. The presented comparison highlights that the potential interactions between the TBC domain of TBC1D17 and Rab5a most likely differ in comparison to the TBC1D20‐Rab1b complex; hence, the determination of the TBC domain structure of TBC1D17 is an important step towards the design of the therapeutics that could target the TBC1D17‐Rab5a interface. Moreover, in the light of our biochemical characterization of the TBC1D17 RBD, the rational design of potential drugs should also take into consideration the interactions between this domain and Rab5a. This analysis provides a rationale for developing peptides as probes or therapeutics that selectively disrupt the TBC1D17‐Rab5a interactions.

The construct encoding RBD designed by Rao et al. is longer at the C‐terminus and covers a fragment from 1 to 305, while the construct tested in this study spans 1–205 amino acids (Rao et al., 2021). We hypothesize that Rao et al. (2021) did not detect the binding of Rab5a with this domain due to possible concealment of the binding residues with labile loops, which are predicted in the AlphaFold model or other secondary structure elements that can be created during Rab protein binding. However, it remains elusive which residues are responsible for direct interaction with Rab5a. Some GAP proteins are recognized to interact with small GTPases through domains other than TBC. For example, SGSM1 (RUTBC2) binds Rab9a by the N‐terminal fragment (amino acids 1–449) covering the RUN domain and the fragment annotated as RBD by InterPro, which was confirmed not only in pull‐down experiments but also by the structural data (PDB ID: 4QXA) (Nottingham et al., 2012; Zhang et al., 2014). Similar results were obtained for SGSM2 (RUTBC1) protein, as the TBC domain does not participate in Rab9A binding. Interestingly, the authors prepared two N‐terminal variants of SGSM1: RUTBC‐RUN (1–185) and RUTBC‐N (1–533), among which only RUTBC‐N (annotated by InterPro in our analysis as RBD) was able to pull down Rab9a, which suggests the importance of this domain (Nottingham et al., 2011). Engagement of domains other than TBC in interactions with Rabs also occurs in proteins which are not close homologs of TBC1D17. RABGAP1 (known also as TBC1D11 or RABCenA) and TBC1D4 interact with Rab36 and Rab2a, respectively, through the PTB domain (Kanno et al., 2010; Tian et al., 2024). However, in earlier experiments the C‐terminal coiled‐coil (CC) fragment of RABGAP1 was preferred by most Rabs (Rab4, Rab6, Rab8, Rab11, Rab14, Rab25, Rab30, Rab33, and Rab37) and the full‐length protein bound only Rab4 and Rab11 (Fuchs et al., 2007). Finally, TBC1D2B containing the PH domain on the N‐terminus, the CC domain in the middle, and the TBC domain on the C‐terminus was able to pull down Rab22a through a protein construct covering the central fragment (141–660 residues), containing the CC fragment (Kanno et al., 2010). These experiments highlight the importance of detailed characterization of other domains than TBC present in GAPs, especially those with unknown functions.

Concerning the cellular context for the observed RBD‐mediated TBC1D17‐Rab5a interactions, we assume that it might be linked to regulation of endolysosomal trafficking which is crucial for maintaining homeostasis in the cell. The membrane of every vehicle is marked with specific Rab GTPases, enabling its identification and determining processing of the cargo (Wandinger‐Ness & Zerial, 2014). To ensure tight and fast endosome regulation and maturation, particular Rab must be effectively activated or replaced with other GTPase. It is well known that active Rabs recruit effectors and thus control vesicular transport. Interestingly, for some GTPases, a mechanism called GEF Rab cascade was observed, in which activation of a particular Rab stimulates recruitment of a GEF protein and activation of other, downstream Rab. Transition of Rab5 to Rab7 is one of the first described GEF cascades, involving Mon1‐Ccz1 complex exhibiting a dual role—Rab5 effector and Rab7 GEF (Borchers et al., 2023; Poteryaev et al., 2010). GEF cascades allow to effectively change the cell membrane protein composition (Rab11 to Rab8 and Rab5 to Rab7 conversion) and regulate pace and direction of the vesicle maturation and cargo delivery (Rab20‐Rab5 regulation) (Borchers et al., 2021, 2023; Pei et al., 2014; Saha et al., 2024). Excessive upregulation of Rab GTPases is balanced with opposite mechanism – Rab GAP cascade, where downstream Rab recruits GAP. The recruited protein does not accelerate or accelerates minimally, GTP hydrolysis in downstream GTPase, but in turn inactivates the upstream Rab. It was first observed for yeast Ypt32p protein (homolog of Rab11 in mammals) which interacts with Gyp1 and promotes inactivation of Ypt1 (homolog of Rab1 in mammals) (Rivera‐Molina & Novick, 2009). Similar mechanism can be observed for Rab5–Rab7 pair in *Caenorhabditis elegans* where TBC‐2 (human TBC1D2 homolog) accumulates on Rab7‐rich late endosomes and inactivates Rab5 (Chotard et al., 2010). It is of note that also RUTBC1 (SGSM2) and RUTBC2 (SGSM1), two of the four Rab GAPs containing RBD according to our research (Figure 6), participate in a cascade involving Rab9a. Both GAPs are recruited by Rab9a and inactivate Rab32 (RUTBC1) or Rab36 (RUTBC2) (Nottingham et al., 2011, 2012). As TBC1D17 is a multidomain protein and, similarly to RUTBC1 and RUTBC2, it contains RBD, we hypothesize that it can participate in Rab GAP cascade. However, according to our knowledge, there is no Rab protein described in the literature that creates a complex with TBC1D17 in their active form and towards which GAP activity is not exhibited. While the first step in GAP cascade assumes preservation of GTP‐bound Rab, our assumption could be confirmed only by the identification of a protein resistant to TBC1D17‐stimulated inactivation.

## 4 CONCLUSIONS

Our structural studies revealed that the TBC domain of TBC1D17, both murine and human, is composed solely of helices and loops, similar to other known TBC domains of GAPs. However, due to the specific orientation of helices within this domain, its overall structure resembles a heart‐like shape, similar to its closest homolog TBC1D15 (Chen et al., 2017), rather than the V‐shaped conformation described for many other TBC domains. Notably, the analysis of the molecules in the asymmetric units of both structures demonstrated that both analyzed structures contained a dimer, which shows that the analyzed TBC domains are able to interact with each other. The dimer interface is present near the conserved residues involved in GTP hydrolysis during interactions with Rabs. Interestingly, we also found such a dimer in the TBC domain structures of TBC1D15. However, due to different annotations of the asymmetric unit, it is formed by the chain A and chain B′ (from the symmetry‐related molecule). The residues located at the dimer interface and involved in intermolecular interactions are relatively conserved.

In addition to structural studies, the interactions between Rab5a and three TBC1D17 fragments, hT17_1‐581_, hT17_268‐581_, and hT17_1‐205_, were analyzed. Interestingly, in the TBC1D17 protein, two domains are annotated in the InterPro database: TBC domain (residues 307–545) and RBD (residues 7–205). Our studies revealed that Rab5a strongly interacts with both TBC1D17 fragments that contained RBD, but the Rab5a interaction with the fragment comprising only the TBC domain was much weaker. The most significant finding of the presented studies is that the previously uncharacterized TBC1D17 RBD is not merely an accessory domain but instead plays a pivotal role in the formation of the Rab5a‐TBC1D17 complex.

## 5 MATERIALS AND METHODS

### 5.1 Cloning, overexpression, and purification of TBC1D17 and Rab5a proteins

The vectors containing full‐length cDNA of mouse Rab5a (UniProt ID: Q9CQD1) and mouse TBC1D17 (UniProt ID: Q8BYH7) proteins were obtained from prof. Yi Ting Zhou (Department of Biochemistry, Zhejiang University School of Medicine, China). Rab5a DNA was re‐cloned to pETM‐11 vector using NcoI and EcoRI restriction enzymes, with simultaneous shortening of the coding sequence to obtain fragment spanning from 16 to 184 amino acid residues (abbreviation used in the article—Rab5a), which is sequentially identical to human Rab5a 16–184. The DNA encoding mouse TBC1D17 TBC domain fragment spanning from 268 to 598 residues (abbreviation used in the article mT17_268‐598_) was amplified by PCR and cloned to pETM‐11 vector using NcoI and XhoI enzymes.

Full‐length cDNA of human TBC1D17 (UniProt ID: Q9HA65) was purchased from Twist Bioscience and was truncated analogously to mouse TBC1D17 gene and cloned by the ligase independent cloning (LIC) (Kim et al., 2011) to pMCSG53 (Midwest Center for Structural Genomics) vector. Moreover, construct encoding Rab‐binding domain (RBD) (residues from 1 to 205, abbreviation used in the article hT17_1‐205_) and the fragment spanning from 1 to 581 amino acid were prepared (abbreviation used in the article hT17_1‐581_). Ultimately, four human constructs were obtained: TBC1D17 1–205 (hT17_1‐205_), TBC1D17 268–581 (hT17_268‐581_), TBC1D17 268–598 (hT17_268‐598_), and TBC1D17 1–581 (hT17_1‐581_). The sequences of the primers used for amplification of desired DNA fragments are presented in Table 3. Fragments of the starters complementary to gene constructs are presented in bold, while remaining parts were added to perform appropriate cloning procedure.

**TABLE 3:** Sequence of the primers used for PCR reactions (fw—forward, rv—reverse).

Rab5a	
fw	**5′** ATATCCATGGCG**AATAAAATATGCCAGTTCAAACTGGTC 3′**
rv	**5′** ATATGAATTCTTA**ATTCTTTGGCAGCTTTTTAGCTATTG 3′**
mT17	
268–598 fw	**5′** ATATCCATGGCG**GAGCTGGGGCAGCGTCC 3′**
268–598 rv	**5′** ATATCTCGAGTTA**TTCTGGCTGTGCCAGCC 3′**
hT17	
268–581 fw 268–598 fw	**5′** TACTTCCAATCCAATGCC**GAATTAGGTCCGCGTCCGACT 3′**
268–581 rv 1–581 rv	**5′** TTATCCACTTCCAATGTTA**ACAAGCAGTCAATTGACGATGTAACGCT 3′**
268–598 rv	**5′** TTATCCACTTCCAATGTTA**AGCAGGTGGCGCTAAGCCCAA 3′**
1–205 fw 1–581 fw	**5′** TACTTCCAATCCAAT**GCCATGGAAGGTGCTGGGTAT 3′**
1–205 rv	**5′** TTATCCACTTCCAATGTTA**ATCGAACAGTTGTAAATGATGAAAAGAATTTGACAA 3′**

*Note:* Nucleotides complementary to the gene are shown in bold.

*Escherichia coli* BL21 Gold competent cells were transformed with prepared vectors and were cultivated on autoinducing LB Broth Base (FORMEDIUM) with kanamycin (50 mg/mL) or ampicillin (150 mg/mL) for 4 h at 37°C with shaking (180 rpm). Then the temperature was lowered to 20°C and cultures were carried out overnight. Next, bacterial cells were separated from the medium by centrifugation at 3700×g for 20 min at 4°C and suspended in binding buffer containing 20 mM HEPES pH 7.5, 500 mM NaCl, 10 mM imidazole, and 1 mM TCEP. Sonication was performed in an ice‐water bath for 4 min in 60 cycles (4 s pulse, 26 s pause). The pellet was separated by centrifugation at 21,000×g for 30 min at 4°C and the supernatant was transferred to a Ni‐NTA chromatographic column, as all TBC1D17 protein variants and Rab5a were expressed with His_6_‐tag. The column was washed with a binding buffer, then with a binding buffer with a high concentration of NaCl (1 M) and again washed with standard binding buffer. His_6_‐tagged proteins were eluted with 20 mL of elution buffer (20 mM HEPES pH 7.5, 500 mM NaCl, 400 mM imidazole, 1 mM TCEP). To remove the excess of imidazole, dialysis was performed against the buffer containing 20 mM HEPES pH 7.5, 500 mM NaCl, and 1 mM TCEP at 4°C, with simultaneous cleavage of His6‐tag using TEV protease. Then, the cut‐off His_6_‐tag was removed from the target proteins by Ni‐NTA chromatography by rinsing twice with binding buffer. Flow through was subsequently concentrated to approximately 2 mL and loaded to HiLoad Superdex 200 16/60 (GE Healthcare) column connected to ÄKTA pure system for size‐exclusion chromatography (SEC). Based on elution volume and known molecular mass, peaks corresponding to target proteins were collected, concentrated, and used for crystallization.

### 5.2 Crystallization and data collection

To establish initial crystallization conditions for TBC1D17 proteins five crystallization screens were tested: Index (Hampton), PEG/Ion (Hampton), BCS (Molecular Dimensions) (Chaikuad et al., 2015), LMB (Molecular Dimensions) (Gorrec, 2016) and Morpheus Fusion (Molecular Dimensions) (Gorrec & Bellini, 2022) using the sitting drop method. The conditions where small protein crystals appeared were optimized using the hanging drop method. The best crystals of mT17_268‐598_ were obtained in condition: 30% Precipitant Mix 4—(20% PEG 3350, 20% PEG 1000, 20% MPD), 50 mM MgCl_2_, 50 mM CaCl_2_, 0.1 M Tris pH 8.5 using hanging drop method, protein concentration 15 mg/mL, and seeding with initial crystals obtained in the screen. The hT17_268‐581_ crystals have grown in condition: 25% PEG Smear Broad, 150 mM Li_2_SO_4_, 0.1 M HEPES pH 7.0, using hanging drop method and protein concentration 6.7 mg/mL. Crystals were cryoprotected with 50% PEG 400 by mixing 1:1 with crystallization condition and frozen in liquid nitrogen. X‐ray diffraction experiment for mT17_268‐598_ was performed in Lund at MAX IV beamline (Ursby et al., 2020) and for hT17_268‐581_ in Hamburg at DESY P13 beamline (Cianci et al., 2017). Data for both proteins were processed in XDSAPP (Sparta et al., 2016) and for hT17_268‐581_ in StarAniso (Tickle et al., 2016) server due to anisotropy of the data.

Prior to molecular replacement, XTRIAGE from the Phenix suite (Liebschner et al., 2019) was used to determine the quality of the data. While no problems were found regarding mT17_268‐598_ structure, it was detected that hT17_268‐581_ structure was twinned with a K, H, −L twin operator. This operator was used in the following stages of refinement.

### 5.3 Structure determination and refinement

The structures of mT17_268‐598_ and hT17_268‐581_ were solved using a molecular replacement method with Phaser (McCoy et al., 2007). For a mT17_268‐598_ chain A of *Sus scorfa* TBC1D15 (PDB ID: 5TUC) (Chen et al., 2017) was used, while hT17_268‐581_ was solved using mT17_268‐598_ as a starting model. The structures were refined manually in Coot (Emsley et al., 2010) and automatically using Refmac5 (mouse protein) (Murshudov et al., 2011) or phenix.refine (human protein) (Afonine et al., 2012). In later refinement stages, TLS parameters were generated with the TLSMD server (Painter & Merritt, 2006).

### 5.4 Oligomeric state analysis of the TBC domain

Analytical size exclusion chromatography for hT17_268‐581_ was performed on Superdex 75 Increase 3.2/300 column (Cytiva). Protein was eluted with PBS buffer at 0.1 mL/min. The results were analyzed with Chromax 2007 (POL‐LAB, Poland) and OriginPro 2022 (OriginLab Corporation, Northampton, MA, USA) software. The size of the protein molecules in solution was determined using a dynamic light scattering (DLS) apparatus Malvern Zetasizer Ultra Red (Malvern Panalytical, Malvern, UK).

### 5.5 Phylogenetic analysis

Using the InterPro database (Blum et al., 2025), a set of 54 human reviewed sequences of GAP‐containing proteins (IPR000195) was downloaded. Due to >98% sequence similarity between TBC1D3 protein paralogs, TBC1D3A (UniProt ID: Q8IZP1) was used as a representative to generate phylogenetic tree and remaining 10 sequences were deleted. Moreover, the sequences of TBC1D28 (UniProt ID: Q2M2D7) and TBC1D29 (UniProt ID: Q9UFV1) proteins were omitted, as it is unclear whether TBC1D28 has GAP activity and TBC1D29 can be a product of pseudogene. Obtained sequences were aligned using MAFFT (Katoh et al., 2018) and based on MSA, a phylogenetic tree was created using IQ‐TREE (version 2.2.2.6) (Minh et al., 2020) using model finder and 1000 replicates of ultrafast bootstrap (Hoang et al., 2018). The final chosen model was Q.insect+R7 (Minh et al., 2021). Model was selected according to Baeysian Information Criterion.

### 5.6 Consurf analysis

Conservation scores of the residues within TBC1D17 proteins were calculated on ConSurf server (Landau et al., 2005; Yariv et al., 2023). To show conservation of amino acids among close homologous proteins, ConSurf searched for close homologs with all default parameters. Analysis with a limited number of sequences was also performed through uploading MSA file used for phylogenetic tree creation with the rest parameters default.

### 5.7 Microscale thermophoresis

Microscale thermophoresis (MST) measurements were performed using a Monolith X instrument (NanoTemper, Germany). His_6_‐Rab5a protein was labeled with the RED‐tris‐NTA 2nd Generation Protein Labeling Kit (NanoTemper, Germany) according to the manufacturer's protocol and subsequently mixed with GTP or GDP at a 1:1 molar ratio. In parallel, 1:1 (v/v) serial dilutions of TBC1D17 variants (hT17_1‐205_, hT17_268‐581_ and hT17_1‐581_), serving as the non‐fluorescent ligands, were prepared in assay buffer (250 mM NaCl, 20 mM HEPES pH 7.5). A constant amount of fluorescently labeled His_6_‐Rab5a (100 nM) was then added to each dilution series. The highest concentrations of unlabeled proteins in the assay were 135 μM for hT17_1‐205_, 405 μM for hT17_268‐581_, and 30 μM for hT17_1‐581_. Samples were loaded into MST premium capillaries and measured at 25°C, with excitation power set to 100%, and medium IR laser power. Dose–response curves were obtained by plotting normalized fluorescence values (Fnorm; fluorescence values after/before IR laser activation) against ligand concentration. Dissociation constants (*K*_d_) were calculated from the binding curve using the law of mass action in MO. Control 2 software (v2.5.4, NanoTemper, Germany). The MST experiment was repeated at least three times for each complex.

## AUTHOR CONTRIBUTIONS

**Dominika Nielipińska:** Writing – original draft, Writing – review & editing, Visualization, Investigation, Validation. **Marta Orlikowska:** Visualization, Investigation. **Maciej Nielipiński:** Investigation, Software. **Bartosz Sekuła:** Validation, Formal analysis. **Katarzyna M. Błażewska:** Validation, Funding acquisition. **Edyta Gendaszewska‐Darmach:** Conceptualization, Supervision, Funding acquisition, Writing – review & editing. **Agnieszka J. Pietrzyk‐Brzezińska:** Conceptualization, Supervision, Validation, Writing – review & editing, Writing – original draft.

## CONFLICT OF INTEREST STATEMENT

The authors declare no conflicts of interest.

## Supporting information


**FIGURE S1.** Comparison of V‐ and heart‐shaped TBC domains. (a) TBC domain of TBC1D1 (yellow, PDB ID: 3QYE) resembles V‐letter. (b) TBC domain of hT17_268‐581_ (light green, this work) resembles the heart. (c) Superposition of TBC1D1 (yellow) and TBC1D17 (green) representing different shapes of TBC domains.
**FIGURE S2:** Oligomeric state analysis of the TBC domain using (a) size‐exclusion chromatography and (b) dynamic light scattering. (a) Chromatogram showing the elution profile of hT17_268‐581_ (solid gold line) compared to a set of protein molecular weight standards (dashed lines): conalbumin (75 kDa), ovalbumin (43 kDa), carbonic anhydrase (29 kDa), ribonuclease A (13.7 kDa), and aprotinin (6.5 kDa). The elution peak of hT17_268‐581_ overlaps with the 75 kDa standard, suggesting a dimeric assembly in solution. Spectra registered at *λ* = 280 nm. (b) Dynamic light scattering data depicting hydrodynamic diameter (*D*_h_) of hT17_268‐581_ with a peak maximum of 8.4 nm. Additionally, the structure of the hT17_268‐581_ dimer is presented next to the plot with a line indicating the approximate width of the crystallographic dimer, measured in Coot.
**FIGURE S3:** Conservation of amino acids in TBC1D17 proteins across TBC‐domain containing GAPs originating from (a) *Mus musculus* or (b) *Homo sapiens*. Blue or green boxes above the sequences represent secondary structure of the proteins. mH1–mH19 marks present helices in murine protein, hH1–hH18 helices in human protein, while remaining parts are unstructured loops. Red rectangles indicate sequential motifs crucial for GAP activity and structural integrity of TBC1D17 protein.
**FIGURE S4:** The MST curves (presenting three independent repetitions) for Rab5a protein interaction with variants of TBC1D17: hT17_1‐205_, hT17_268‐581_ and hT17_1‐581_.

## Data Availability

Structure factors and coordinates were deposited in the Protein Data Bank with the following accession numbers: 9SI6 (murine TBC domain of TBC1D17) and 9SI7 (human TBC domain of TBC1D17).
