# Supplementary material for: Structural and functional insights into TBC1D17 highlight the importance of the previously uncharacterized Rab‐binding domain
Source: Protein Sci. 2026 Apr 17;35(5):e70581. doi: 10.1002/pro.70581 (PMC13090580; doi:10.1002/pro.70581)
Supplement: Supplementary file 1 — FIGURE S1. Comparison of V‐ and heart‐shaped TBC domains. (a) TBC domain of TBC1D1 (yellow, PDB ID: 3QYE) resembles V‐letter. (b) TBC domain of hT17268‐581 (light green, this work) resembles the heart. (c) Superposition of TBC1D1 (yellow) and TBC1D17 (green) representing different shapes of TBC domains. FIGURE S2: Oligomeric state analysis of the TBC domain using (a) size‐exclusion chromatography and (b) dynamic light scattering. (a) Chromatogram showing the elution profile of hT17268‐581 (solid gold line) compared to a set of protein molecular weight standards (dashed lines): conalbumin (75 kDa), ovalbumin (43 kDa), carbonic anhydrase (29 kDa), ribonuclease A (13.7 kDa), and aprotinin (6.5 kDa). The elution peak of hT17268‐581 overlaps with the 75 kDa standard, suggesting a dimeric assembly in solution. Spectra registered at λ = 280 nm. (b) Dynamic light scattering data depicting hydrodynamic diameter (D h) of hT17268‐581 with a peak maximum of 8.4 nm. Additionally, the structure of the hT17268‐581 dimer is presented next to the plot with a line indicating the approximate width of the crystallographic dimer, measured in Coot. FIGURE S3: Conservation of amino acids in TBC1D17 proteins across TBC‐domain containing GAPs originating from (a) Mus musculus or (b) Homo sapiens. Blue or green boxes above the sequences represent secondary structure of the proteins. mH1–mH19 marks present helices in murine protein, hH1–hH18 helices in human protein, while remaining parts are unstructured loops. Red rectangles indicate sequential motifs crucial for GAP activity and structural integrity of TBC1D17 protein. FIGURE S4: The MST curves (presenting three independent repetitions) for Rab5a protein interaction with variants of TBC1D17: hT171‐205, hT17268‐581 and hT171‐581. [file PRO-35-e70581-s001.docx]

**SUPPLEMENTARY DATA**

**FIGURES**


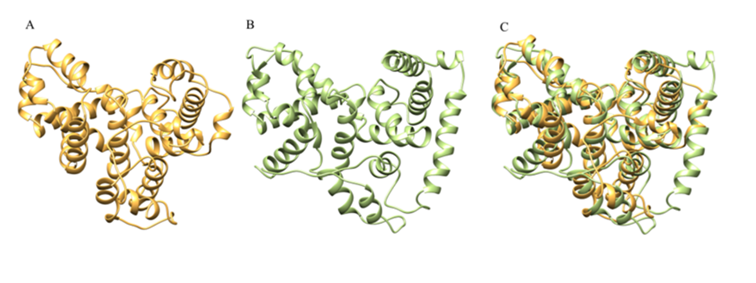


**Fig. S1. Comparison of V- and heart-shaped TBC domains. (A)** TBC domain of TBC1D1 (yellow, PDB ID: 3QYE) resembles V-letter. **(B)** TBC domain of hT17_268-581_ (light green, this work) resembles the heart. **(C)** Superposition of TBC1D1 (yellow) and TBC1D17 (green) representing different shapes of TBC domains.

**
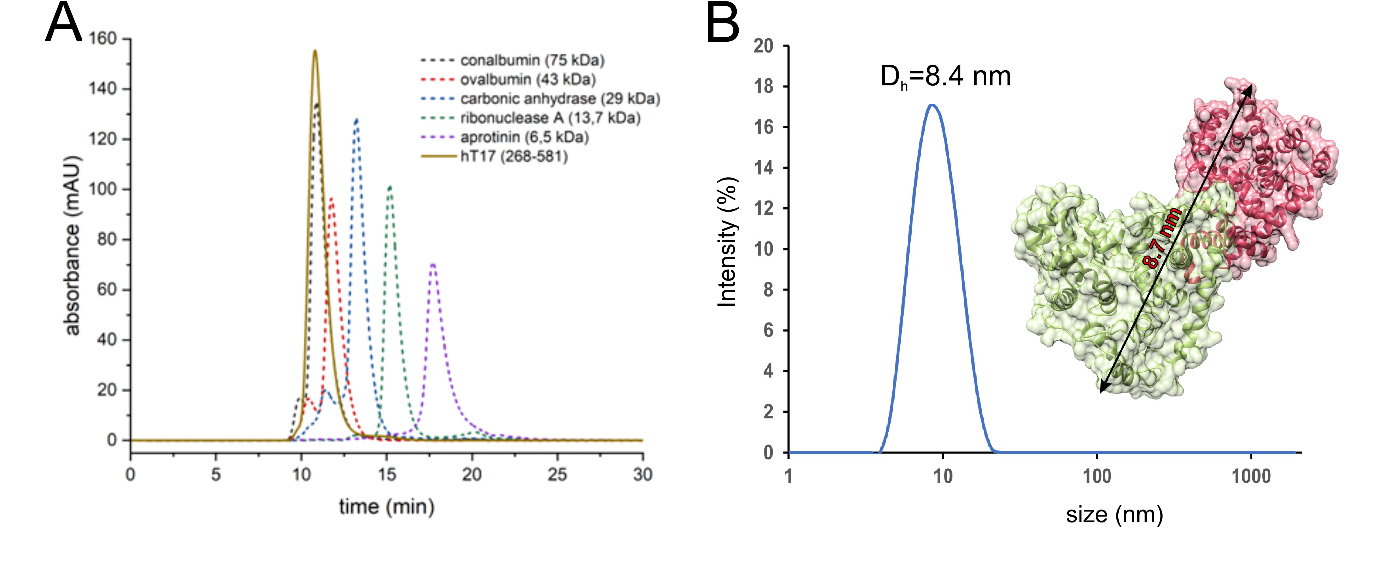
**

**Fig. S2.** **Oligomeric state analysis of the TBC domain using (A) size-exclusion chromatography and (B) dynamic light scattering.** **(A)** Chromatogram showing the elution profile of hT17_268-581_ (solid gold line) compared to a set of protein molecular weight standards (dashed lines): conalbumin (75 kDa), ovalbumin (43 kDa), carbonic anhydrase (29 kDa), ribonuclease A (13.7 kDa), and aprotinin (6.5 kDa). The elution peak of hT17_268-581_ overlaps with the 75 kDa standard, suggesting a dimeric assembly in solution. Spectra registered at λ = 280 nm. **(B)** Dynamic light scattering data depicting hydrodynamic diameter (D_h_) of hT17_268-581_ with a peak maximum of 8.4 nm. Additionally, the structure of the hT17_268-581_ dimer is presented next to the plot with a line indicating the approximate width of the crystallographic dimer, measured in Coot.


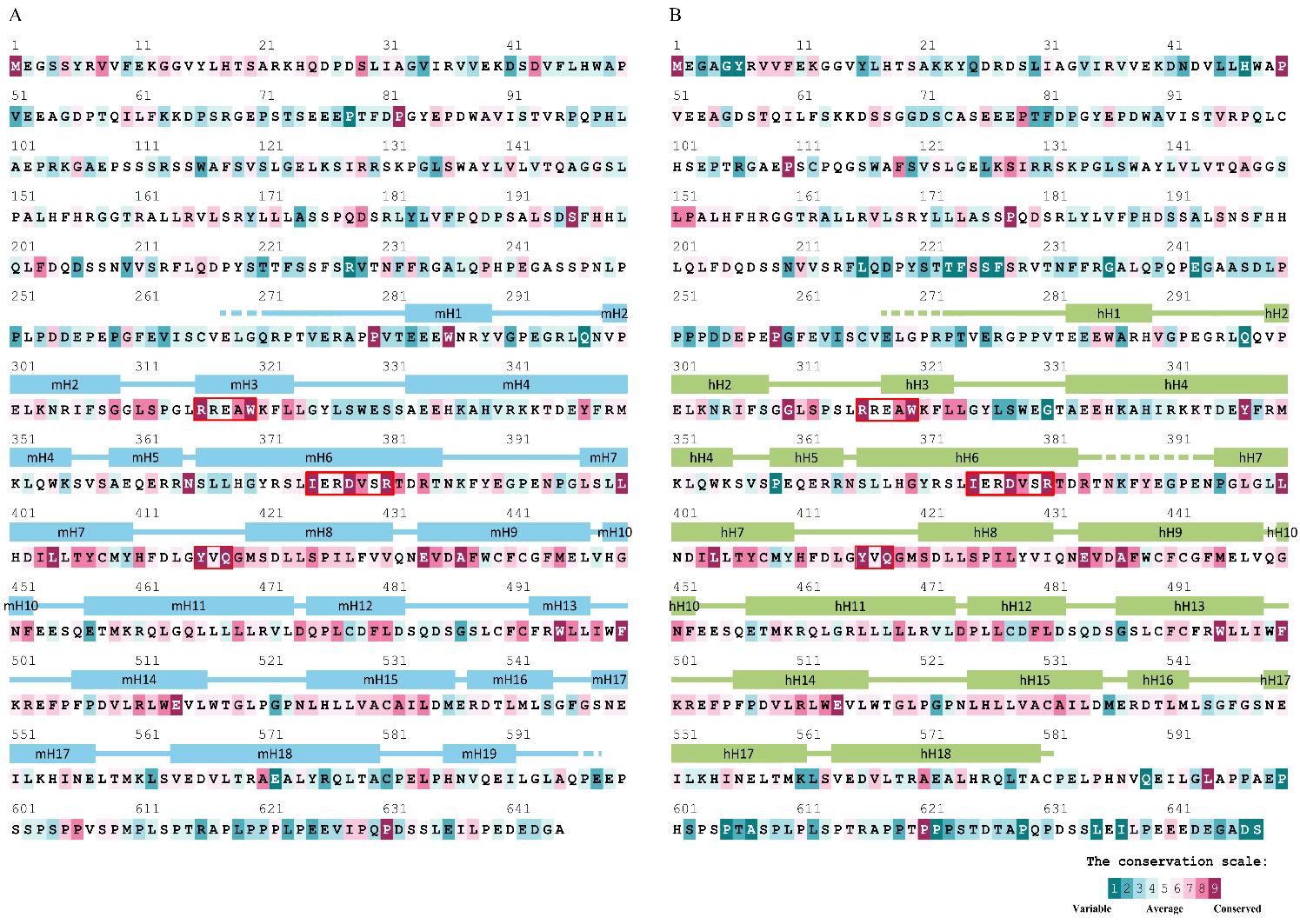


**Fig. S3.** **Conservation of amino acids in TBC1D17 proteins across TBC-domain containing GAPs originating from (A) *Mus musculus*or (B) *Homo sapiens*.** Blue or green boxes above the sequences represent secondary structure of the proteins. mH1-mH19 marks present helices in murine protein, hH1-hH18 helices in human protein, while remaining parts are unstructured loops. Red rectangles indicate sequential motifs crucial for GAP activity and structural integrity of TBC1D17 protein


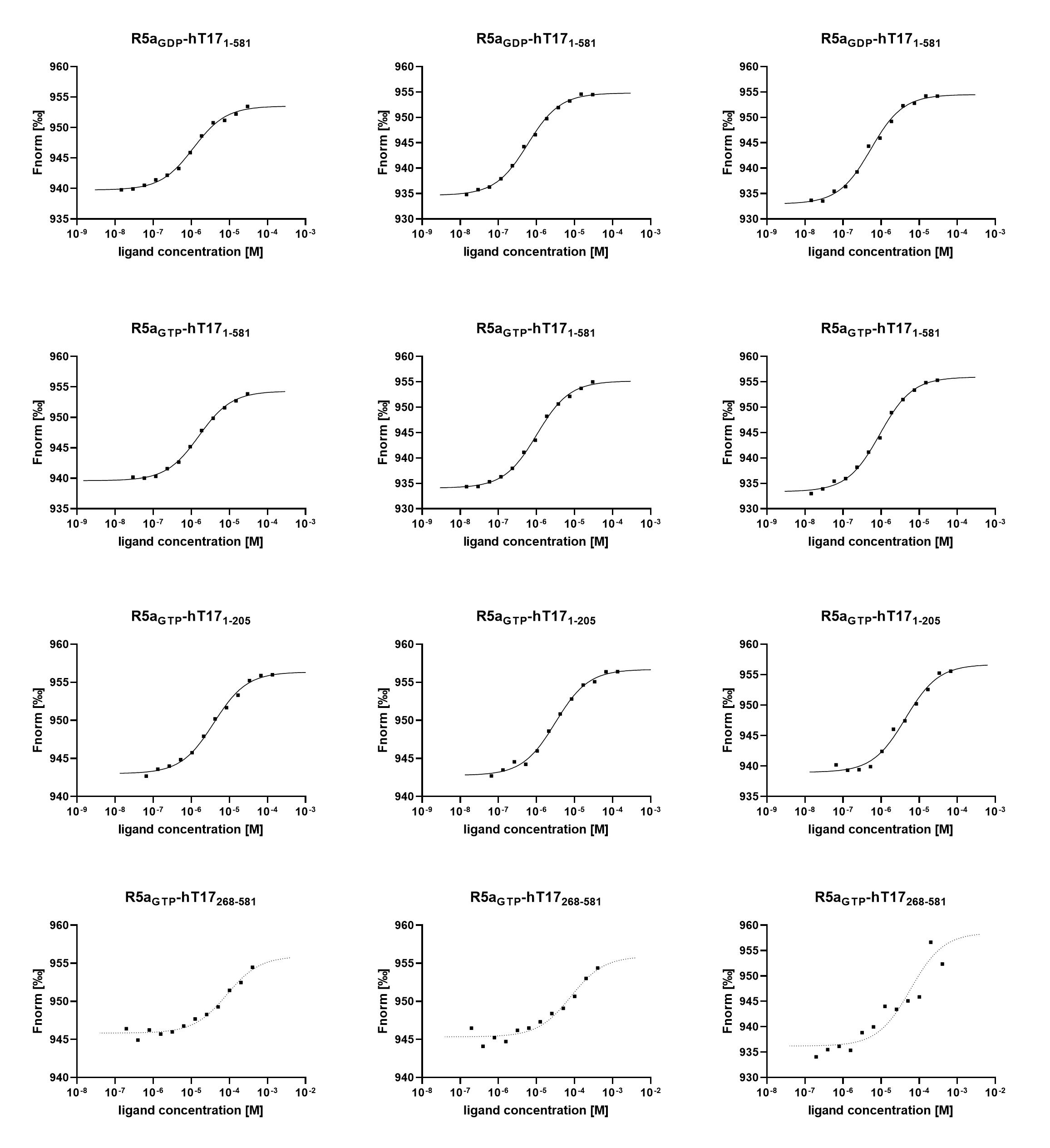


**Fig. S4.**The MST curves (presenting three independent repetitions) for Rab5a protein interaction with variants of TBC1D17: hT17_1-205_, hT17_268-581_ and hT17_1-581_.
